# Post-treatment with Ma-Huang-Tang ameliorates cold-warm-cycles induced rat lung injury

**DOI:** 10.1038/s41598-017-00459-3

**Published:** 2017-03-22

**Authors:** Meng-Meng Xiao, Chun-Shui Pan, Yu-Ying Liu, Li-Qian Ma, Li Yan, Jing-Yu Fan, Chuan-She Wang, Rong Huang, Jing-Yan Han

**Affiliations:** 1 Department of Integration of Chinese and Western Medicine, School of Basic Medical Sciences, Peking University, Beijing, 100191 China; 2 Tasly Microcirculation Research Center, Peking University Health Science Center, Beijing, 100191 China; 3 Key Laboratory of Microcirculation, State Administration of Traditional Chinese Medicine of the People’s Republic of China, Beijing, 100191 China; 4 Key Laboratory of Stasis and Phlegm, State Administration of Traditional Chinese Medicine of the People’s Republic of China, Beijing, 100191 China

## Abstract

Frequent and drastic ambient temperature variation may cause respiratory diseases such as common cold and pneumonia, the mechanism for which is not fully understood, however, due to lack of appropriate animal models. Ma-Huang-Tang (MHT) is widely used in China for treatment of respiratory diseases. The present study aimed to investigate the effect of MHT on temperature alternation induced rat lung injury and explore underlying mechanisms. Male Sprague-Dawley rats were exposed to a cold environment for 1 h and then shifted to a warm environment for 30 min. This cold and warm alteration cycled 4 times. Rats were administrated with MHT (1.87 g/kg) by gavage 6 h after cold-warm-cycles. Cold-warm-cycles induced pulmonary microcirculatory disorders, lung edema and injury, decrease in the expression of tight junction proteins, increase in VE-cadherin activation, increase in the expression and activation of Caveolin-1, Src and NF-κB, and NADPH oxidase subunits p47^phox^, p40^phox^ and p67^phox^ membrane translocation and inflammatory cytokines production. All alterations were significantly ameliorated by post-treatment with MHT. This study showed that rats subjected to cold-warm-cycles may be used as an animal model to investigate ambient temperature variation-induced lung injury, and suggested MHT as a potential strategy to combat lung injury induced by temperature variation.

## Introduction

Living body is exposed to environment and responds to any alteration in the environment by modulating physiological function. It is commonly accepted that an environment including ambient temperature that changes too frequently and drastic, may overwhelm the adaptive capacity of living body thus causes disease. It was reported that temperature variation is significantly associated with emergency admissions for stroke and myocardial infarction^1, 2^. Respiratory diseases often occur in daily temperature variation, such as season shift, day-night cycle or indoor/outdoor alternation in winter^3, 4^. A time-series study published recently found that daily and multiday temperature variation may increase respiratory hospitalizations^5^. Ambient temperature variation provoked respiratory diseases differ significantly, ranging from common cold to pneumonia, depending on the individual affected. An effective strategy remains in need in clinic to relieve the temperature variation-provoked respiratory disease or prevent it from aggravation^6–9^.

The mechanism for the temperature variation-induced respiratory diseases is far from clear. Previous studies have demonstrated that simple cold stimulation can cause respiratory symptoms. Inhalation of cold air has been reported to cause contraction of tracheal smooth muscle and decreased lung perfusion^1^, increased levels of inflammatory cytokines IL-1, IL-6, IL-8 and TNF-α *in vivo* ^10^ and *in vitro* ^11^. Cold stimulation was found to enhance the expression of P-selectin and β2 integrin CD11b/CD18^12, 13^, promoting leukocytes adhering to microvessels. However, no study has been published so far to investigate the mechanism underlying the respiratory diseases induced by temperature variation, but not only simple cold, in animal model.

The impact of ambient temperature variation on health has long been recognized in traditional Chinese medicine, and Ma-Huang-Tang (MHT), a Chinese medicine formula, was formulated to cope with temperature variation-caused disorders, including fever, headache, and cough. MHT is widely used in China for treatment of respiratory diseases including influenza, upper respiratory tract infection, acute and chronic bronchitis and asthma^14, 15^. MHT is composed of herba ephedrae (HE), ramulus cinnamomi (RC), semen armeniacae amarum (SAA) and radix glycyrrhizae (RG). Studies in mice showed that MHT inhibits ovalbumin-induced increase in IFN-γ level in bronchoalveolar lavage fluid (BALF) and eosinophilia in lung tissue^16^, decreases the virus titers in both nasal and BALF and increases the anti-influenza virus IgM, IgA and IgG antibody titer in serum and BALF^17^. However, the role and underlying mechanisms of MHT in temperature variation-caused lung injury have not been explored.

The purpose of the present study was two fold. (1) To establish an animal model in which cold-warm-cycle was performed to imitate ambient temperature variation challenge to induce lung injury and gain insight into the underlying mechanisms; (2) MHT was applied to treat the cold-warm-cycles-induced lung injury with attempt to clarify the effect and mechanism.

## Results

### MHT decreases the number of leukocytes adherent to pulmonary venules

**Figure 1:**
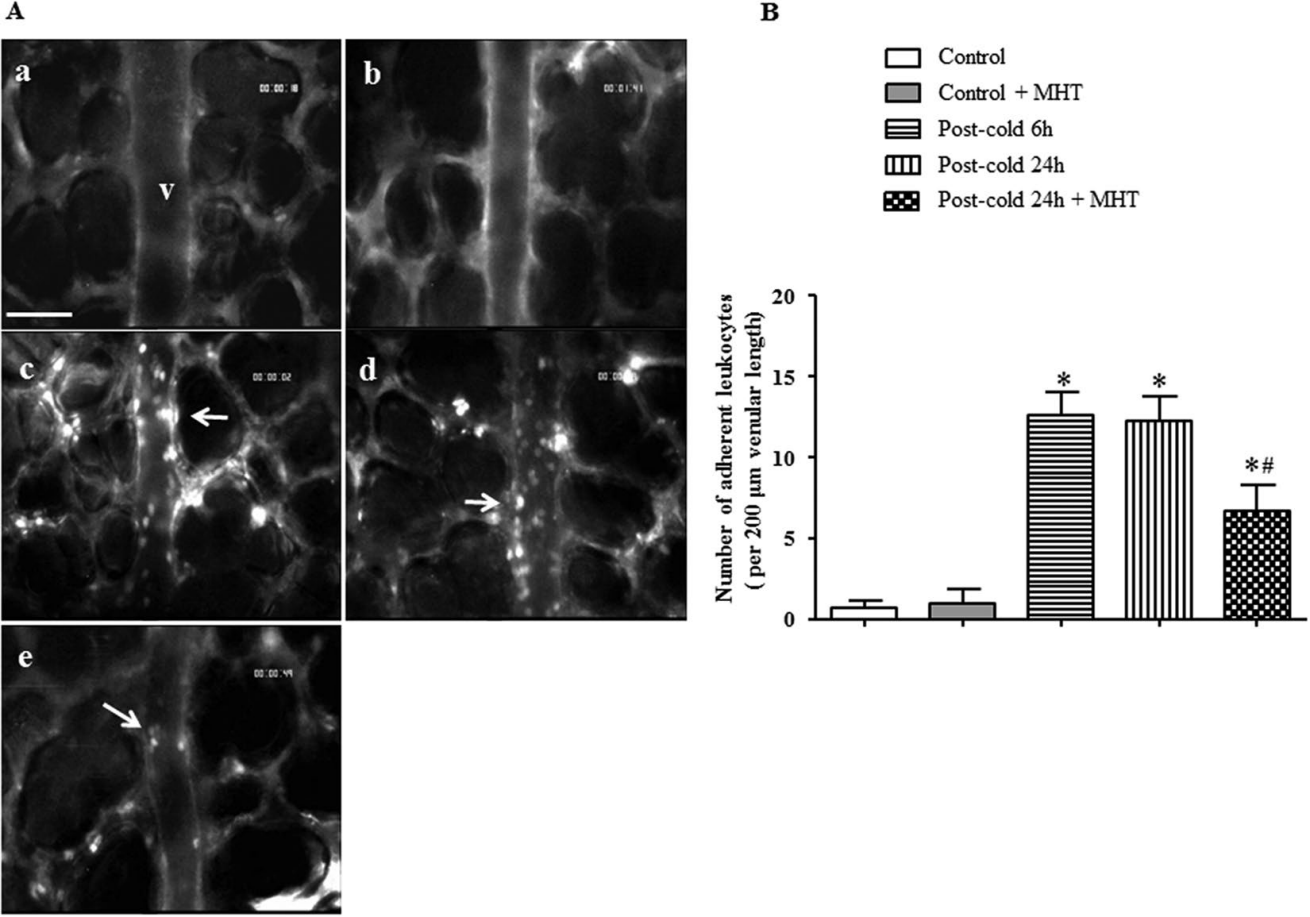
Effect of MHT post-treatment on leukocyte adhesion to pulmonary venules after cold-warm-cycles. (**A**) Representative images of rat pulmonary venules in Control (a), Control + MHT (b), Post-cold 6 h (c), Post-cold 24 h (d), and Post-cold 24 h + MHT (e) group, respectively. Arrows indicate adherent leukocytes. Bar = 50 μm. (**B**) Statistical result of the number of leukocytes adherent to the pulmonary venules of rats in different groups. N = 6. Results are presented as mean ± SE. *P < 0.05 vs. Control group; ^#^P < 0.05 vs. Post-cold 24 h group.

Warm-cold-cycles-caused lung injury was characterized by microcirculatory disturbance and inflammatory reaction. To address the effect of MHT post-treatment on cold-warm-cycles-induced pulmonary microcirculation disturbance, the leukocyte adhesion to lung venules was observed with an intravital fluorescent microscope. Figure 1A shows the representative images of pulmonary venules in different groups. In Control (Fig. 1A, a) and Control + MHT (Fig. 1A, b) group, few leukocytes adherent to the pulmonary venules were observed, while in Post-cold 6 h group (Fig. 1A, c) and Post-cold 24 h (Fig. 1A, d) group there were numerous leukocytes that adhered to the pulmonary venules. Post-treatment with MHT attenuated leukocyte adhesion to the pulmonary venules (Fig. 1A, e) 24 h after cold-warm-cycles. Figure 1B is the quantification of the adherent leukocytes to the pulmonary venules of 200 μm length in different groups, which confirmed the results from survey above.

### MHT reduces inflammatory cell infiltration in the lung tissue induced by cold-warm-cycles

**Figure 2:**
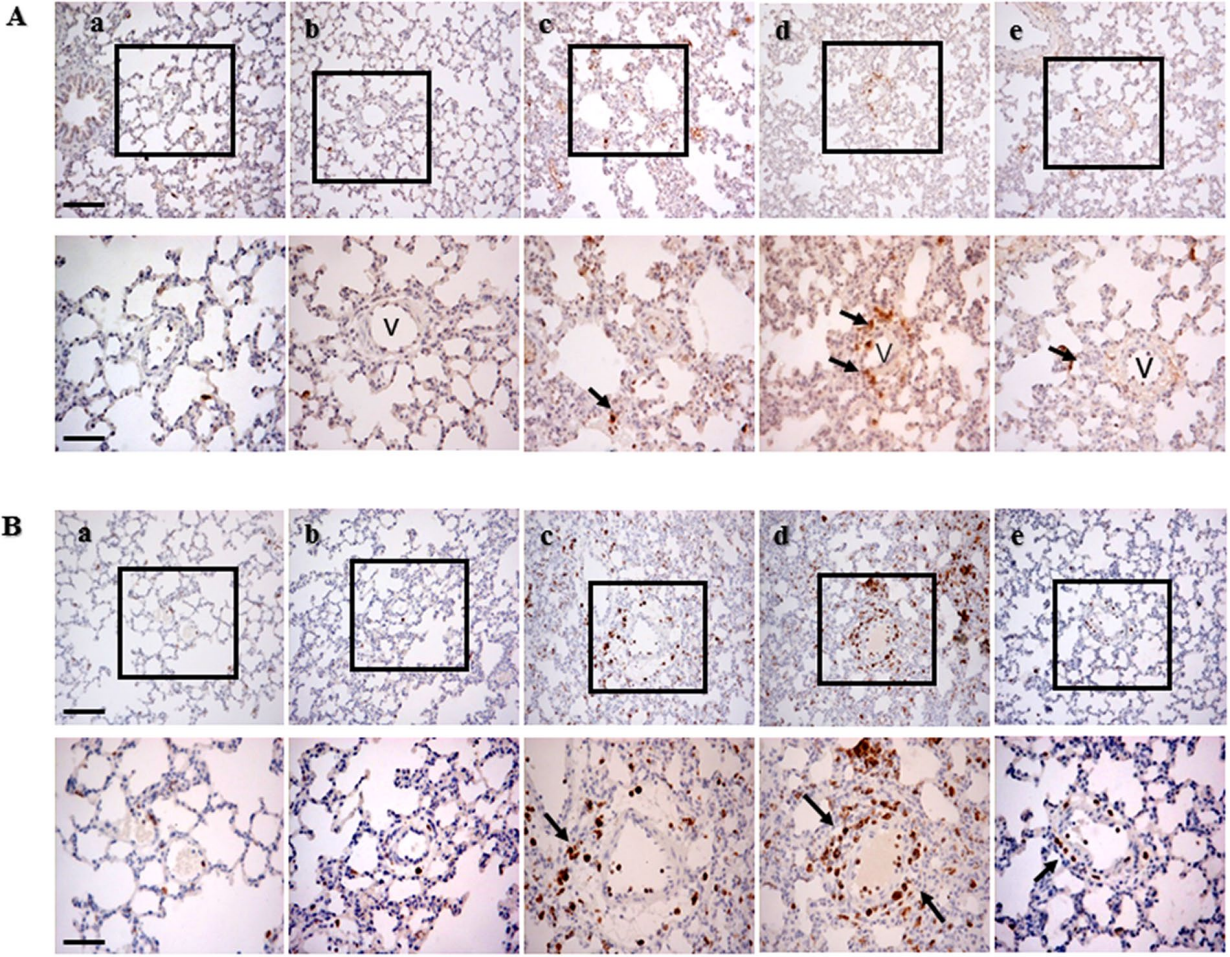
Effect of MHT post-treatment on infiltration of pulmonary neutrophils and macrophages. (**A**) Representative images of immunohistochemical staining for MPO to reveal neutrophils in Control (a), Control + MHT (b), Post-cold 6 h (c), Post-cold 24 h (d), Post-cold 24 h + MHT (e) group, respectively. Arrows indicate MPO positive cells. Bar = 100 μm. The area within the rectangle in each image in upper panel is enlarged and presented below. Bar = 50 μm. (**B**) Representative images of immunohistochemical staining for CD68 to reveal macrophages in Control (a), Control + MHT (b), Post-cold 6 h (c), Post-cold 24 h (d), Post-cold 24 h + MHT (e) group, respectively. Arrows indicate CD68 positive cells. v, microvessel. Bar = 100 μm (upper panel) and 50 μm (lower panel).

To examine the effect of MHT post-treatment on inflammatory cell infiltration caused by cold-warm-cycle, MPO-positive neutrophils and CD68-positive macrophages were examined by immunohistochemistry staining and the images are presented in Fig. 2A and B, respectively. Compared with Control (Fig. 2A, a) and Control + MHT (Fig. 2A, b) group, numerous MPO-positive cells were observed in the interstitial tissue in Post-cold 6 h (Fig. 2A, c) group and Post-cold 24 h (Fig. 2A, d) group. Similar result was observed in immunohistochemistry staining of CD68-positive macrophages (Fig. 2B), showing that cold-warm-cycles provoked inflammatory cell infiltration. In post-treatment with MHT group, the number of infiltrating neutrophils and macrophages was significantly reduced (Fig. 2A, e and B, e), suggesting the ameliorating effect of MHT on inflammatory cell infiltration.

### MHT ameliorates cold-warm-cycles-induced lung edema and lung injury

**Figure 3:**
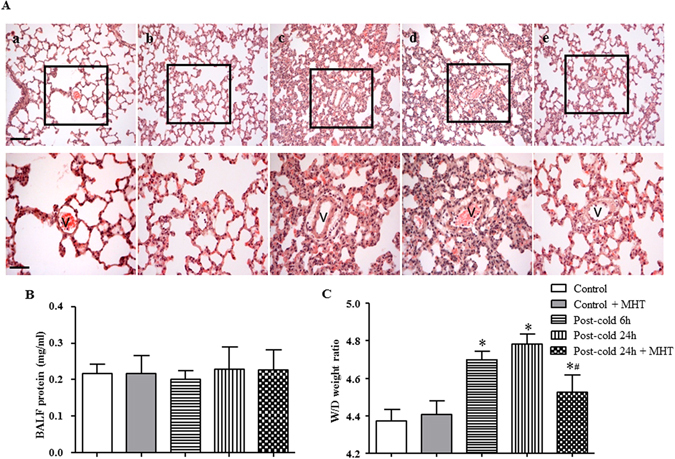
Effect of MHT post-treatment on histology of the lung tissue and lung edema. (**A**) Representative H&E staining images of rat lung tissue in Control (a), Control + MHT (b), Post-cold 6 h (c), Post-cold 24 h (d), Post-cold 24 h + MHT (e) group, respectively. Bar = 100 μm. The area within the rectangle in each image in upper panel is enlarged and presented below. v, microvessel. Bar = 50 μm. (**B**) BALF protein concentration in different groups. No difference was noted among groups. (**C**) Lung W/D weight ratio in different groups. N = 4–6. Results are presented as mean ± SE. *P < 0.05 vs. Control group; ^#^P < 0.05 vs. Post-cold 24 h group.

Cold-warm-cycles caused lung edema and lung histological change, as shown in Fig. 3A. An obvious thickening of the alveolar wall, inflammatory cell infiltration as well as perivascular edema were observed in H&E staining images of Post-cold groups (Fig. 3A, c and d) but not in Control (Fig. 3A, a) group and Control + MHT (Fig. 3A, b) group. Cold-warm-cycles induced lung edema was confirmed by the increase in W/D weight ratio of lung tissue (Fig. 3C). However, BALF protein concentration remained nearly consistent in all groups (Fig. 3B). Of notice, post-treatment with MHT ameliorated these cold-warm-cycles caused manifestations significantly (Fig. 3A, e and C), suggesting the protective effect of MHT on cold-warm-cycles-induced lung edema and injury.

To explore the respective contribution of the 4 ingredients of MHT to the observed reliving effect on lung injury, histologic examination was also conducted for each of the ingredients contained in MHT. The results showed that all ingredients but RG exhibited reliving effect on the cold-warm-cycles-elicited lung injury, although to a different extent (Supplementary Figure S1, e through h).

### MHT prevents the degradation of tight junction proteins and activation of adherent junction protein induced by cold-warm-cycles

**Figure 4:**
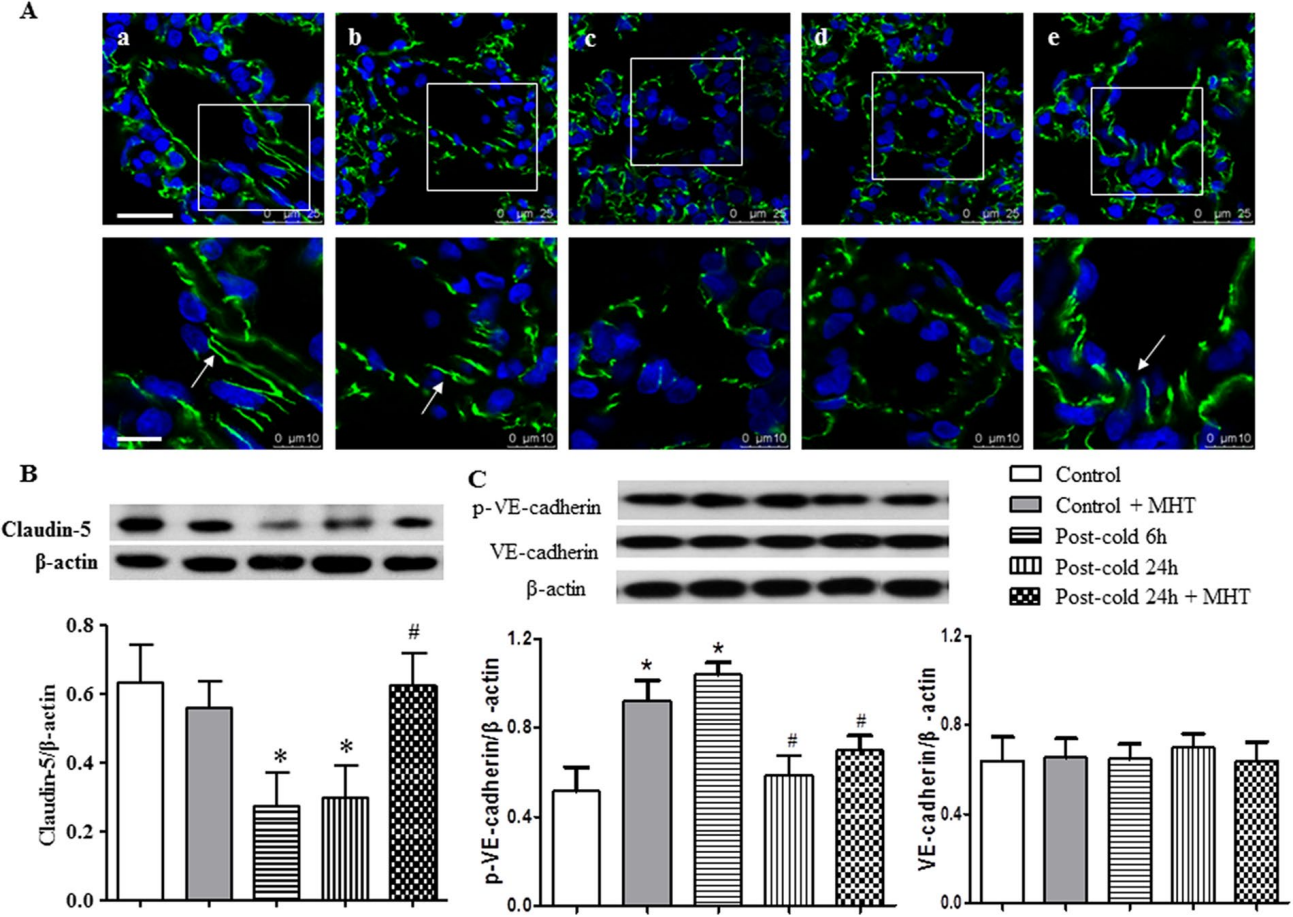
Effect of MHT post-treatment on the expression of Claudin-5 and activation of VE-cadherin in the rat lung tissues. (**A**) Representative immunofluorescence confocal images of rat lung tissue in Control (a), Control + MHT (b), Post-cold 6 h (c), Post-cold 24 h (d), and Post-cold 24 h + MHT (e) group, respectively. Bar = 25 μm. The area within the rectangle in each image in upper panel is enlarged and presented below. Bar = 10 μm. The sections were immunochemically stained for Claudin-5 (green). (**B**) Western blot for the expression of Claudin-5 in different groups with the quantification of Claudin-5 showing below. (**C**) Western blot for the VE-cadherin in different groups with the quantification of Claudin-5 showing below. N = 4. Results are presented as mean ± SE. *P < 0.05 vs. Control group; ^#^P < 0.05 vs. Post-cold 24 h group.

To explore the rationale behind the protective effect of MHT on lung edema, the expressions of tight junction proteins were assessed by immunohistochemistry and western blot. Immunofluorescence staining of Claudin-5 showed that, compared with Control group (Fig. 4A, a) and Control + MHT group (Fig. 4A, b), the expression of Claudin-5 was decreased in the lung tissues 6 h and 24 h after cold-warm-cycles (Fig. 4A, c and d). Claudin-5 reduction was obviously relieved by post-treatment with MHT (Fig. 4A, e). The results of immunohistochemistry were confirmed by the western blot analysis (Fig. 4B). Adherent junction is known to play a critical role as well in regulation of microvessel permeability in lungs, thus, VE-cadherin, a major adherent junction protein, was evaluated by western blot. The result revealed no difference in the expression of VE-cadherin among groups, though, but a significant increase in p-VE-cadherin after cold-warm-cycles, which was attenuated by MHT treatment (Fig. 4C), indicating the involvement of adherent junction in NHT action.

**Figure 5:**
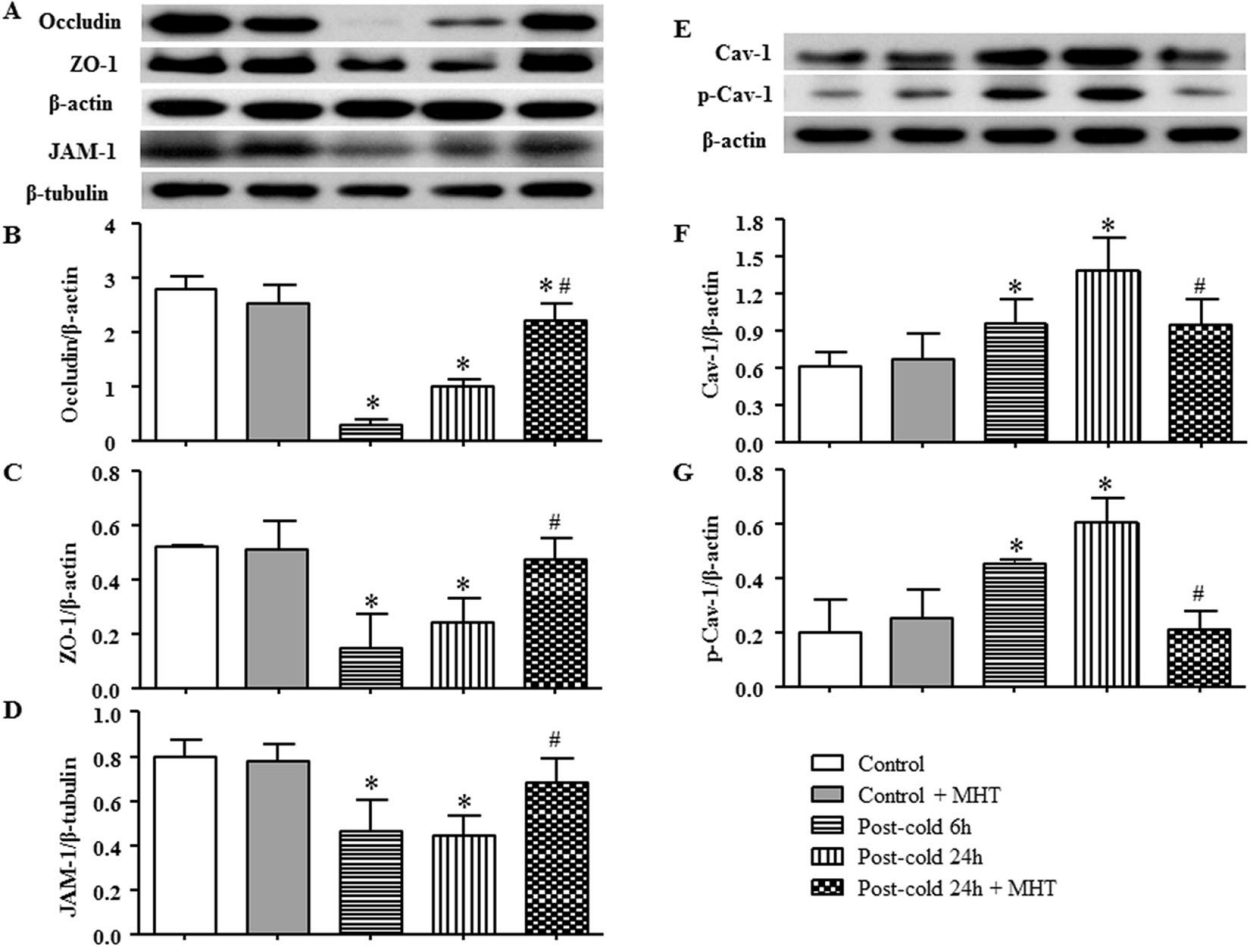
Effect of MHT post-treatment on the expression of tight junctions Occluding, ZO-1 and JAM-1, and the activation of Caveolin-1 in rat lung tissue. (**A**) The representative western blot bands of tight junctions Occluding, ZO-1 and JAM-1 in different groups. (**B**) Quantification of expression of Occluding; (**C**) Quantification of expression of ZO-1; (**D**) Quantification of expression of JAM-1; N = 4. (**E**) The representative western blot bands of Cav-1 and p-Cav-1 in different groups; (**F**) Quantification of expression of Cav-1; (**G**) Quantification of expression of p-Cav-1; N = 4. Results are presented as mean ± SE. *P < 0.05 vs. Control group; ^#^P < 0.05 vs. Post-cold 24 h group.

Occluding, JAM-1 and ZO-1, the other three tight junction proteins, were also examined by western blot to assess their expressions in the lung tissues from different groups. As shown in Fig. 5, a similar pattern of variations was observed among groups, indicating that cold-warm-cycles led to degradation of tight junction proteins, which was ameliorated by post-treatment with MHT. Taking together, these results suggested that MHT ameliorated cold-warm-cycles-elicited pulmonary microvessel hyperpermeability, at least in part, by regulating the paracellular pathway.

To explore the respective contribution of the 4 ingredients of MHT to the observed reliving effect on junction proteins, western blot was performed for each ingredient as compared with MHT. The result showed that the effects of MHT are mostly attributable to HE and RC and less to SAA, but nearly has nothing to do with RG (Supplementary Figures S4–S6).

### MHT inhibits the expression and phosphorylation of Caveolin-1 induced by cold-warm-cycles

Caveolae are organelles in endothelial cells which mediate microvascular permeability via transcellular transport, a process that initiates with Caveolin-1 phosphorylation^18^. We explored the expression and phosphorylation of Caveolin-1 in the lung tissues from various groups by western blot. As shown in Fig. 5E, F and G, cold-warm-cycles challenge evoked a time-dependent increase in both expression and phosphorylation of Caveolin-1 compared with Control and Control + MHT group, indicating that Caveolin-1 was involved in cold-warm-cycles-induced microvessel hyperpermeability. Interestingly, the increased expression and phosphorylation of Caveolin-1 were inhibited significantly by post-treatment with MHT, suggesting transcellular transport as another pathway for MHT to ameliorate microvessel hyperpermeability.

### MHT inhibits the phosphorylation of Src and NF-κB nuclear translocation

**Figure 6:**
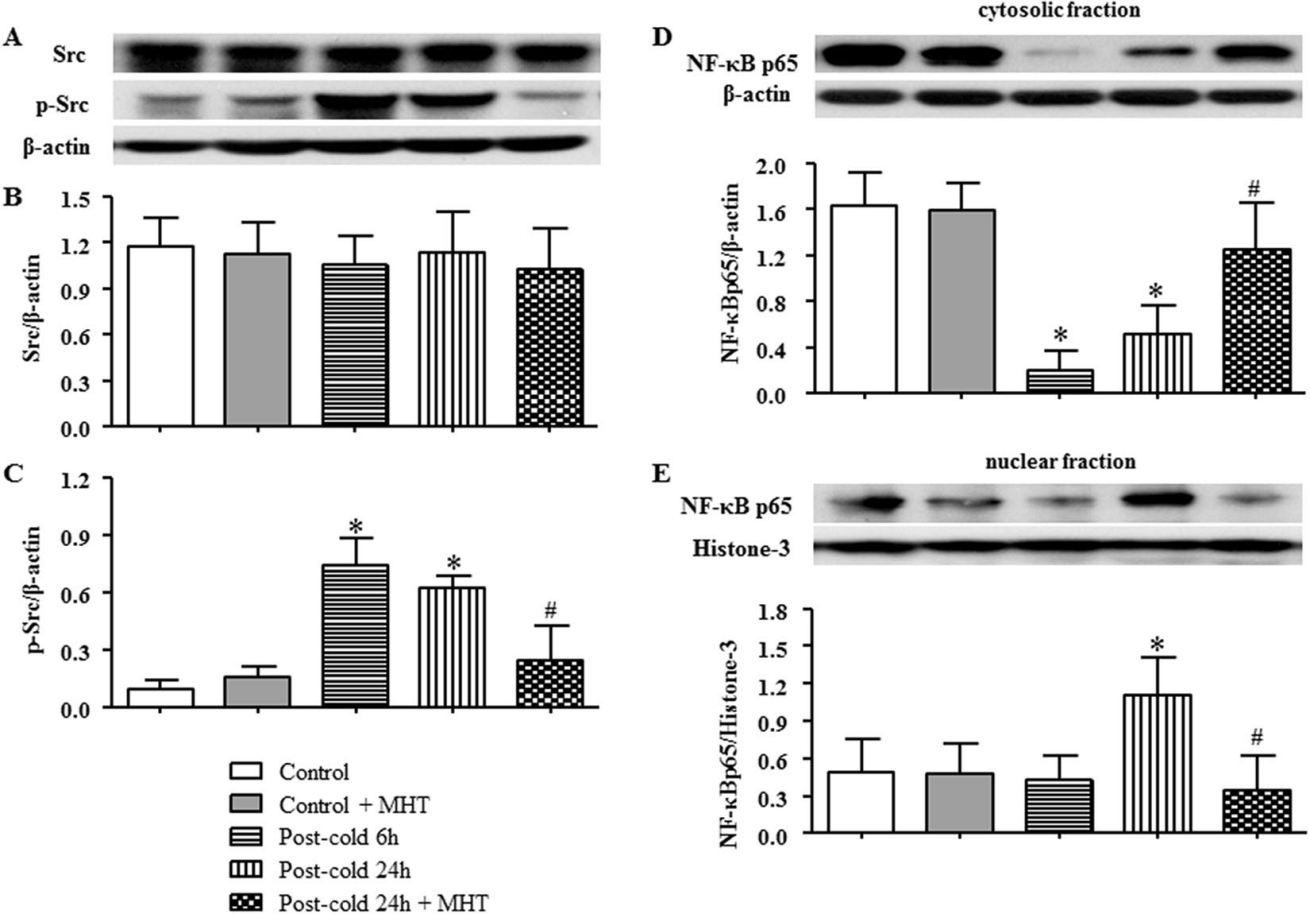
Effect of MHT post-treatment on the activation of Src and nuclear translocation of NF-κB p65. (**A**) The representative western blot bands of Src and p-Src in different groups. (**B**) Quantification of expression of Src; (**C**) Quantification of expression of p-Src; (**D**) The expression of NF-κB p65 in cytosolic fraction; (**E**) The expression of NF-κB p65 in nuclear fraction; N = 4. Results are presented as mean ± SE. *P < 0.05 vs. Control group; ^#^P < 0.05 vs. Post-cold 24 h group.

Src is known as the upstream signaling molecule involved in the regulation of microvessel permeability^18–20^. And NF-κB plays a critical role in the progression of inflammatory process^2^ by nuclear translocation of NF-κB p65 initiating transcription of inflammatory mediators. Thus, western blot analysis was undertaken to assess the expression and phosphorylation of Src and nuclear translocation of NF-κB p65 in the lung tissues from different groups. The results revealed that cold-warm-cycles induced an increase in phosphorylation (Fig. 6A and C), but not expression, of Src (Fig. 6A and B), as well as nuclear translocation of NF-κB p65 (Fig. 6D and E), all of which were blunted significantly by post-treatment with MHT, suggesting involvement of Src activation and nuclear translocation of NF-κB p65 in attenuating effect of MHT on microvessel hyperpermeability induced by cold-warm-cycles.

The effect of MHT ingredients on NF-κB p65 was assessed and compared with MHT per se, revealing that NF-κB p65 nuclear translocation after cold-warm-cycles was prevented by HE to an extent similar to MHT. RC protected NF-κB p65 from nuclear translocation as well, but to a much less extent. Again, SAA and RG did not exert effect (Supplementary Figure S2 and Table S1).

### MHT reduces pro-inflammatory cytokine level in rat lung tissue, BALF and blood plasma

**Figure 7:**
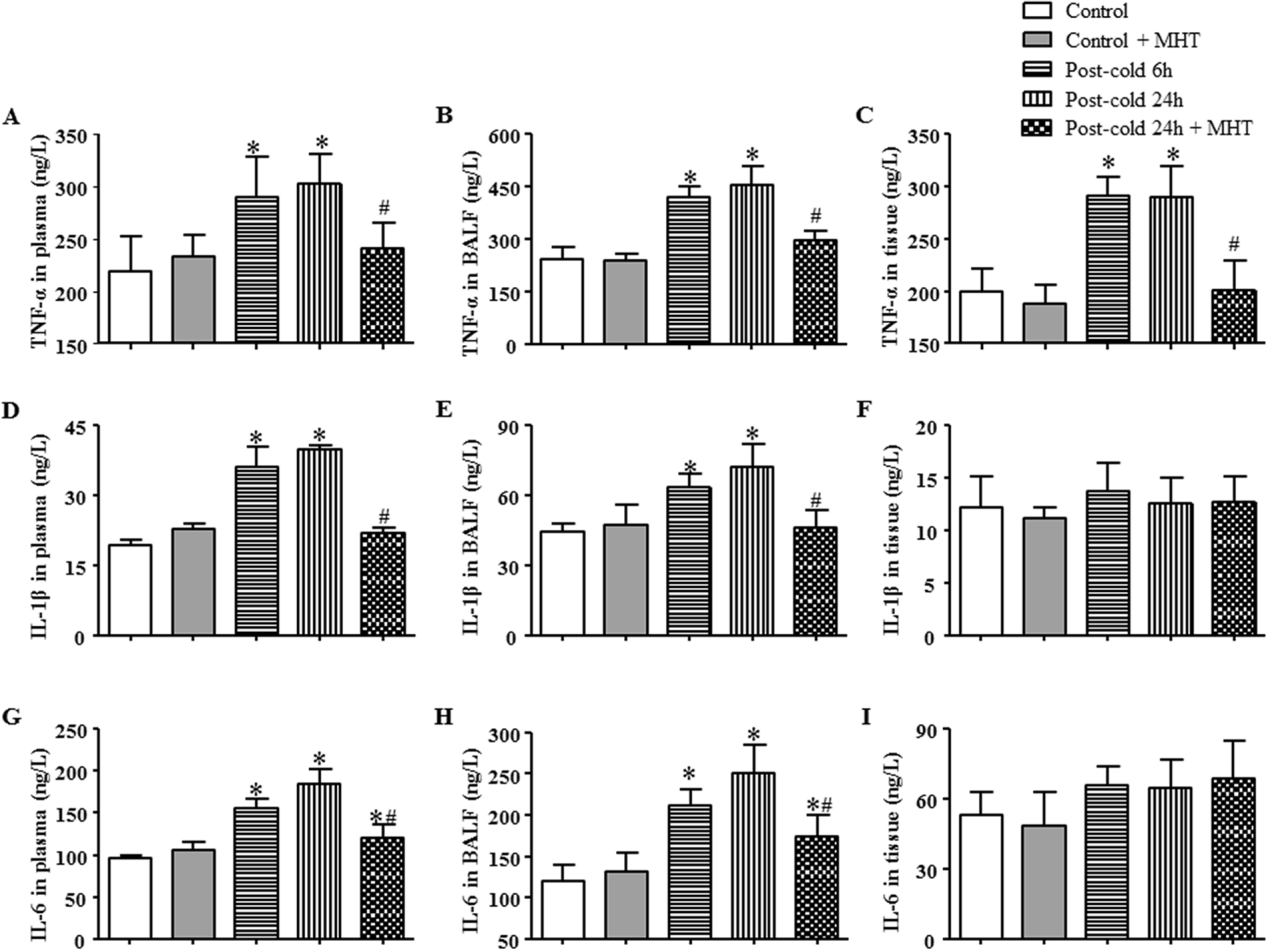
Effect of MHT on the levels of TNF-α, IL-1β and IL-6 in plasma, BALF and lung tissue. (**A**) The concentration of TNF-α in rat plasma from different groups; (**B**) The concentration of TNF-α in rat BALF from different groups; (**C**) The concentration of TNF-α in rat lung tissue from different groups; (**D**) The concentration of IL-1β in rat plasma from different groups; (**E**) The concentration of IL-1β in rat BALF from different groups; (**F**) The concentration of IL-1β in rat lung tissue from different groups; (**G**) The concentration of IL-6 in rat plasma from different groups; (**H**) The concentration of IL-6 in rat BALF from different groups; (**I**) The concentration of IL-6 in rat lung tissue from different groups. The concentrations of cytokines were determined by ELISA. Results are presented as mean ± SE. *P < 0.05 vs. Control group; ^#^P < 0.05 vs. Post-cold 24 h group.

Activation of NF-κB leads to release of inflammatory cytokines aggravating lung injury^21^. We thus further detected the level of inflammatory cytokines TNF-α, IL1-β, IL-6 and IL-10 to address the inflammatory process induced by cold-warm-cycles. The results showed that cold-warm-cycles significantly increased the levels of TNF-α, IL-1β and IL-6 in rat blood plasma as well as in BALF (Fig. 7A,B,D,E,G and H). In rat lung tissue, on the other hand, the level of TNF-α was increased (Fig. 7C), while the levels of IL1-β and IL-6 remained unchanged after cold-warm-cycles (Fig. 7F and I). The level of IL-10 did not alter in plasma, nor in BALF (data not shown). All the increased TNF-α, IL1-β and IL-6 were attenuated significantly by post-treatment with MHT.

### MHT attenuates activation of NADPH oxidase induced by cold-warm-cycles

**Figure 8:**
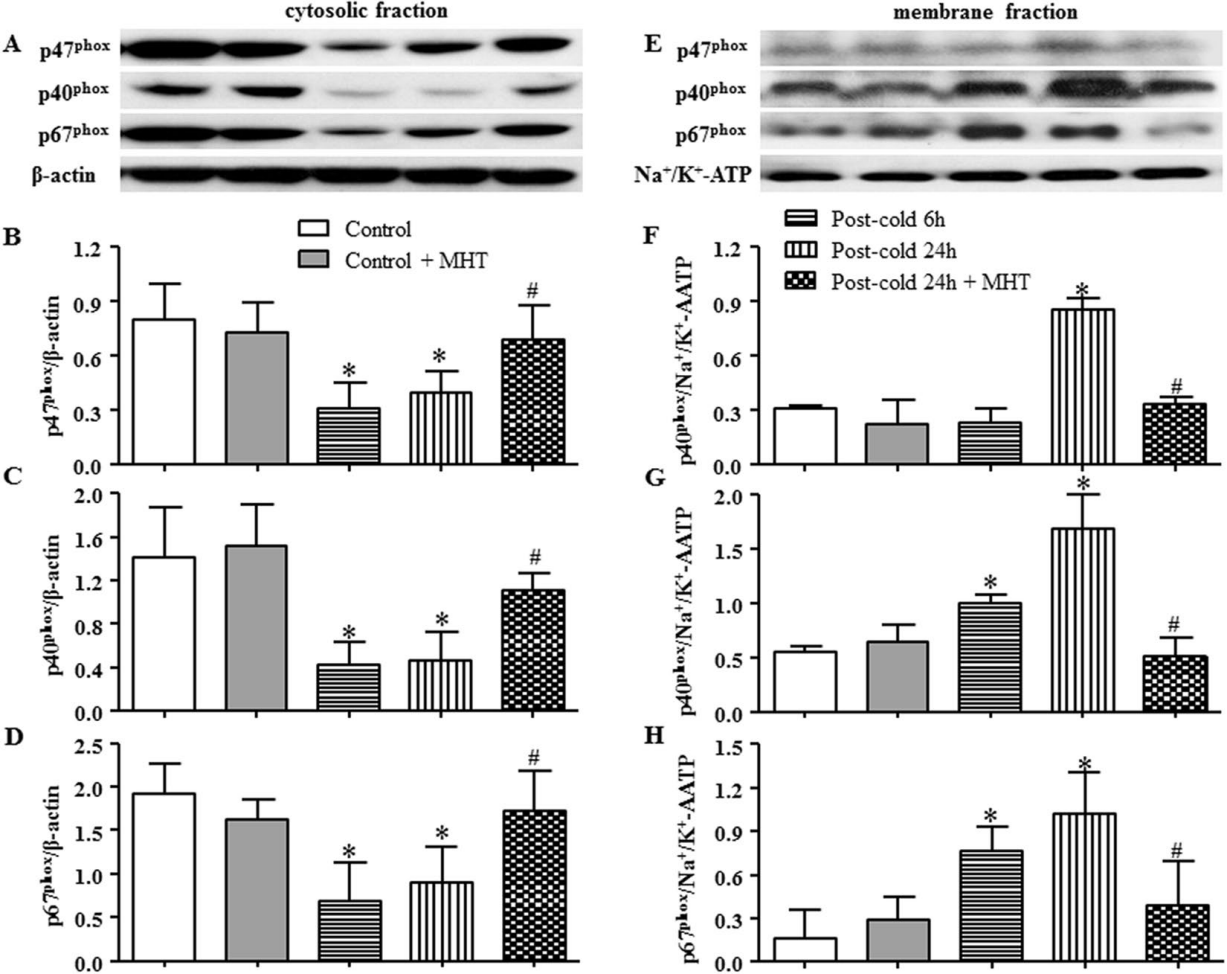
Effect of MHT post-treatment on membrane translocation of NADPH oxidase subunits p47^phox^, p40^phox^ and p67^phox^. (**A,E**) The representative western blot bands of NADPH oxidase subunits p47^phox^, p40^phox^ and p67^phox^ in cytosolic fraction and membrane fraction, respectively; (**B**) Quantification of p47^phox^ in cytosol; (**C**) Quantification of p40^phox^ in cytosol; (**D**) Quantification of p67^phox^ in cytosol; (**F**) Quantification of p47^phox^ in cell membrane; (**G**) Quantification of p40^phox^ in cell membrane; (**H**) Quantification of p67^phox^ in cell membrane; N = 4. Results are presented as mean ± SE. *P < 0.05 vs. Control group; ^#^P < 0.05 vs. Post-cold 24 h group.

In response to inflammatory stimulation, leukocytes adhere to venular walls and, meanwhile, become activated and produce massive peroxide through NADPH oxidase^22^. To assess the activation of NADPH oxidase, its organizer and modulator subunits p47^phox^, p40^phox^ and p67^phox^ were detected by western blot. As compared to Control and Control + MHT group, the expression of p47^phox^ (Fig. 8A and B), p40^phox^ (Fig. 8A and C) and p67^phox^ (Fig. 8A and D) was significantly decreased in cytosolic fraction in response to cold-warm-cycles stimulation, and correspondingly, increased in membrane fraction (Fig. 8E,F,G and H), suggesting that cold-warm-cycles was able to induce the translocation and activation of NADPH oxidase. These alternations in expression of NADPH oxidase subunits in both cytoplasm and cell membrane were significantly blunted by post-treatment with MHT, indicating the potential of MHT to attenuate the oxidative stress imposed by cold-warm-cycles.

The protected effect on membrane translocation of NADPH oxidase was assessed for each ingredient relative to MHT, and found that HE and RC exerted a similar effect as MHT did, but to a less extent, while SAA and RG exhibited no effect (Supplementary Figure S3 and Table S1).

## Discussion

The present study demonstrated that exposure to cold (−15 °C 1 h)-warm (25 °C 30 min)-cycles for 4 times was able to induce lung injury in rat, manifesting microcirculatory disorder, lung tissue edema, and elevated level of inflammatory cytokines along with activation of Src, NF-κB and NADPH oxidase. Interestingly, all the alterations were attenuated by MHT treatment, suggesting the potential of this Chinese medicine in therapy of temperature variation induced lung injury and providing insight for understanding the underlying mechanism.

Previous studies reported that cold exposure induces expression of P-selectin and leukocyte adhesion molecules CD11b/CD18^12, 13^ and infiltration of neutrophils and macrophages in airways^10^, suggesting likely occurrence of pulmonary microcirculatory disorders in response to cold challenge. The present study provided direct evidence showing an obvious leukocyte adhesion to pulmonary venules after cold-warm-cycles. This leukocyte adhesion was observed 6 h after cold-warm-cycles, and maintained by 24 h after cold-warm-cycles. The exact time remains to be determined for this disorder to initiate and terminate if without interference. Leukocyte adhesion to microvascular walls is known as a critical episode in the pathophysiology of cold injury^23–25^, as well as of lung injury imposed by systematic inflammation. Adherent leukocytes may in turn be activated and release oxidants and proteases, which damage microvascular walls resulting in serum albumin efflux, perivascular neutrophil infiltration in acute phase and macrophage infiltration in sub-acute phase^3^. Consistent with these results, the present study observed MPO-positive neutrophils and CD68-positive macrophages infiltration around blood vessels, in addition to leukocytes adhesion to pulmonary venules, indicating an increase in microvascular permeability after cold-warm-cycles. It is likely that the impact imposed by cold-warm-cycles in the present model, possibly as well as by the prompt ambient temperature variation in clinic involves microvascular disturbance, albumin efflux, inflammatory cells infiltration and pulmonary interstice edema. On the other hand, direct injury of respiratory tract epithelium was not observed in the present setting, as shown by the lack of difference in BALF protein content among the groups. The reason for this result is at present unknown. Moreover, a 100 percent survival rate was observed for animals from all groups by 7 days after cold-warm-cycles, suggesting that the stimulation in the present animal model is not strong enough to threaten the animal life, at least over the period of observation.

There are two pathways responsible for the regulation of microvascular permeability: paracellular pathway mediated by intercellular junctions, and transcellular pathway via Caveolae^26–28^. The present study showed that both pathways were implicated in the increase in microvascular permeability after cold-warm-cycles in the present setting, as evidenced by the decrease in tight junction proteins and increase in p-VE-cadherin along with an increased Caveolin-1 expression and activation. These results highly suggest activation of a protein that mediates both para- and trans-cellular pathways. We speculated that this protein is Src, because phosphorylation of Src has been reported not only to increase the expression and phosphorylation of Caveolin-1^4^ but also reduce Occluding and ZO-1 from tight junctions^29–31^. This speculation was validated by the finding that Src in rat pulmonary tissue was activated in response to cold-warm-cycles challenge. Importantly, all the insults induced by cold-warm-cycles were relieved by treatment with MHT, indicating the potential of this Chinese medicine to attenuate cold-warm-cycles-provoked pulmonary microcirculatory disorder involving inactivation of Src.

Src localizes in the middle of inflammatory signaling pathway in pulmonary diseases^2^. On the one hand, Src can be activated by a wide range of mediators, including peroxide derived from NADPH oxidase following exposure to inflammatory stimuli^5^, and, on the other hand, participates in regulation of diverse proteins, including NF-κB triggering transcription of inflammatory cytokines^32, 33^. Interestingly, the present study revealed that cold-warm-cycles evoked activation not only of Src, but also of NADPH oxidase and NF-κB, as well as release of inflammatory cytokines. These results imply that in the animal model of present study, the lung injury caused by cold-warm-cycles manifests not only microcirculatory disorders, but rather a typical inflammation process. Moreover, the result that MHT ameliorated all the insults showed the potential of MHT to counteract temperature variation-caused pulmonary inflammation. Nevertheless, the detailed mechanism thereby MHT exerts effect on the cold-warm-cycles-induced lung injury needs to be elucidated by further studies.

MHT is a compound Chinese medicine containing 4 ingredients, experiment was performed in the present study to evaluate the role of each ingredient in cold-warm-cycles-caused lung injury (Supplementary data). Among the 4 ingredients, HE, RC and SAA each alone was found effective in restoration of cold-warm-cycles-induced lung injury in terms of inflammatory cells infiltration and lung tissue edema when tested by histology. Assessment of signaling pathway revealed that HE and RC may interfere in NF-κB p65 nuclear translocation and NADPH oxidase membrane tanslocation after cold-warm-cycles, as MHT did. While SAA had no influence on the pathways tested. These results suggested that HE, RC and SAA all contributed to the observed effect of MHT on inflammation, although, but most likely via different signaling pathway. On the other hand, RG did not show any effect on the variables tested in current study. This result is not a surprise, given the fact that RG is almost always applied as an adjuvant in Chinese medicine formulation. Nevertheless, the exact contribution of each ingredient in MHT to its protective effect on cold-warm-cycles-induced lung injury needs clarification by further study.

The present study has some limitations. For example, ambient temperature variation may cause disorder in respiratory system in any season as soon as this variation is severe and/or frequent enough, such as moving from an air conditioned environment to outdoor in summer. Even in winter, the temperature difference between indoor and outdoor varies depending on the location. The animal model used in the present study is too simple to simulate all the conditions. Moreover, although obvious microcirculation disturbance and inflammation were observed following cold-warm-cycles, no evidence is detected showing occurrence of injury of respiratory tract epithelium, the reason for which remains to be identified by further study.

In conclusion, this study demonstrated that as an ambient temperature variation model, cold-warm-cycles evoked rat lung injury manifesting microcirculatory disorders and pulmonary inflammation concurring with activation of Src, NADPH oxidase and NF-κB, all of which were attenuated by treatment with MHT. This result verifies the potential of MHT as a therapy for ambient temperature variation induced lung injury in clinic by elucidating the underlying mechanism for its effect.

## Materials and Methods

### Animals

Male Sprague-Dawley rats weighing 180–200 g were obtained from the Animal Center of Peking University Health Science Center. The certificate code number was SCXK 2006–0008. The animals were housed at 24 ± 1 °C and relative humidity of 50 ± 1% with a 12 h light/dark cycle and given standard laboratory diet and water. The animals were fasted for 12 h before experiment but free to access to water. The experimental procedures were in accordance with the European commission guideline (2010/63/EU). All animals were handled according to the guidelines of the Peking University Animal Research Committee. The experimental protocol was approved by the Committee on the Ethics of Animal Experiments of Peking University Health Science Center (LA2015143).

### Regents

MHT granules, consisted of HE granule (39.4%), RC granule (26.3%), SAA granule (15.5%) and RG granule (18.8%), were provided by Guangdong Yi Fang Pharmaceutical Co Ltd (Guangzhou, China). Rhodamine 6G was from Fluka Chemie AG (Buchs, Switzerland). Antibodies against Cav-1, phosphor-Cav-1, Src, phosphor-Src, p-VE-cadherin, β-actin, β-tubulin and Histone-3 were purchased from Cell Signaling Technology (Beverly, MA, USA). Antibodies against JAM-1, Claudin-5, ZO-1, NF-κB p65, Na+/K+-ATPase, NADPH oxidase subunit p47phox and p40phox, VE-cadherin were purchased from Santa Cruz Biotechnology (Santa Cruz, CA, USA). Antibodies against Occluding, NADPH oxidase subunit p67phox were purchased from Abcam (Cambridge, UK).

### Experiment protocols

Rats were randomly assigned into one of five groups: (1) Control group, (2) Control + MHT group, (3) Post-cold 6 h group, (4) Post-cold 24 h group, and (5) Post-cold 24 h + MHT group. The number of animals in each group for assessment of various parameters is detailed in Table 1. The rats were exposed to a cold environment (−15 °C) for 1 h and then shifted to a warm environment (25 °C) for 30 min. This cold-warm alternation cycled 4 times to imitate environment temperature alternation, and then the rats were maintained in room temperature. Six hours later, the rats were subjected to determination of various parameters (Post-cold 6 h group) or administrated by gavage with MHT suspension (1.87 g/kg, Post-cold 24 h + MHT group) or normal saline (Post-cold 24 h group) and subjected to determination of parameters 24 h after transferring to room temperature. Rats of Control groups were kept at 25 °C for 6 h without food and water delivered, and then transferred to room temperature, administrated 6 h later by gavage with MHT suspension (1.87 g/kg, Control + MHT group) or normal saline (Control group). All of the parameters of the rats in Control groups were determined 24 h after transferring to room temperature.

**Table 1:** Number of animals for different experimental groups and various parameters.

	Control	Control + MHT	Post-cold 6 h	Post-cold 24 h	Post-cold 24 h + MHT	Total
Survival rate	8	8	8	8	8	40
Leukocyte adhesion	6	6	6	6	6	30
HE and immunohistology staining	6	6	6	6	6	30
Wet/Dry ratio	6	6	6	6	6	30
BALF protein and cytokines concentration in plasma, BALF, lung tissue	6	6	6	6	6	30
Western Blot	(4)	(4)	(4)	(4)	(4)	
Total	32	32	32	32	32	160

The same animals were used for determination of survival rate. The same animals were used for determination of H&E and immunohistochemistry staining. The same animals were used for determination of BALF protein and the cytokine concentrations in plasma, BALF and lung tissue, as well as for western blot analysis. Control, Control group; Control + MHT, Control group plus MHT post-treatment; Post-cold 6 h, Post-cold 6 h group; Post-cold 24 h, Post-cold 24 h group; Post-cold 24 h + MHT, Post-cold 24 h group plus MHT post-treatment.

### Leukocyte adhesion to the pulmonary venules

Microcirculation parameters were assessed as previously described^34, 35^. Six or twenty four hours after cold-warm-cycles, the rats were anesthetized with 20% urethane (2.0 g/kg body weight, i.m.). The rats were placed in supine position, submitted to tracheotomy and ventilated with a positive-pressure respirator (ALC-V8, Shanghai, China). The chest was opened between the third and fifth ribs to expose the left lung lobe. During the observation, the ventilator was turned off while the ventilation tube was infused with continuous air to maintain positive airway pressure so that the lung surface beneath a thin glass plate was expanded enough to keep stable. The lung was superfused continuously by warm (37 °C) normal saline. Each observation lasted approximately 30 seconds and after that the ventilator was opened again for the rats to breath. The lung microcirculation was observed with an upright intravital fluorescent microscope (BX51WT, Olympus, Tokyo, Japan) in combination with a supersensitive charge-coupled device camera (USS-301, UNIQ Vision, Santa Clara, CA, USA) using a helium-neon laser beam for illumination.

To observe the leukocyte adhesion to pulmonary venules, the rats were administrated with fluorescence tracer Rhodamine 6G (0.1 mg/kg) via the femoral vein. Venules with diameter ranging from 30 to 50 μm and length of 200 μm were selected for observation. The leukocytes that maintained motionless for more than 30 seconds were defined as the adhering leukocytes, and the number of adhering leukocytes was counted.

### Histological and immunohistochemical staining

Histologic and immunohistochemical examinations were carried out as previously described^35–37^. In brief, 6 or 24 h after cold-warm-cycles, rat right middle lung lobe was excised, fixed in 4% paraformaldehyde in 0.1 M phosphate buffer solution (pH 7.4), and processed for paraffin sections using an automated processing unit (RM2255, Leica, Berlin, Germany). The sections of 5 μm of pulmonary specimens for histology were stained by hematoxylin and eosin (H&E). The images were captured by a digital camera connected to a microscope (BX512DP70, Olympus, Tokyo, Japan). The sections for immunohistochemistry were incubated with rabbit polyclonal antibody against MPO (1:200, Thermo Scientific, Fremont, CA, USA), rabbit antibody against CD68 (1:50, Abcam, Cambridge, UK) or mouse antibody against Claudin-5 (1:50, Santa Cruz Biotechnology, Santa Cruz, USA) after blocked with bovine serum albumin, and then incubated with a biotinylated secondary antibody from an avidin-biotin-complex-peroxidase kit or a fluorescent secondary antibody. Positive staining was revealed by reacting with diaminobenzidine (BD Biosciences Pharmingen, CA, USA) or a laser scanning confocal microscope (TCS SP5, Leica, Mannheim, Germany).

### Evaluation of pulmonary edema

In another set of experiments, the lung wet-to-dry (W/D) weight ratio and protein level in BALF were determined for the animals in each group 6 or 24 h after cold-warm-cycles. For lung W/D ratio, the upper lobe of right lung of rat was excised and weighed followed by drying at 80 °C for 72 h^34, 35^. The ratio of the wet lung weight to the dry lung weight was calculated. For determination of protein concentration of BALF, the rats were inserted with a plastic cannula into the trachea and douched with 2 mL sterile saline (pH 7.4) with withdrawing for three times. BALF samples were centrifuged (1580 g, 4 °C) for 15 min and the supernatants were collected^35, 36^. The concentration of protein in the supernatants of BALF was quantitated by bicinchoninic acid assay method (Thermo Scientific, Fremont, CA, USA).

### Concentration of inflammatory cytokines

At 6 or 24 h after cold-warm-cycles, the rats were sacrificed; blood was collected from the abdominal aorta of each rat and anticoagulated with heparin (20 U/mL whole blood). The BALF was collected as previous described^34, 35^ and the left lung was excised and homogenized. The blood plasma and BALF were centrifuged (Allegra 64R Centrifuge, Beckman Coulter, Berlin, Germany), respectively, at 1580 g for 15 min at 4 °C and stored at −20 °C. The levels of TNF-α, IL-6, IL-1β and IL-10 in plasma, BALF and lung tissue homogenate were determined by using ELISA kits (Andygene, Beijing, China) according to the manufacturer’s instructions.

### Western blotting assay

Rats were sacrificed 6 or 24 h after cold-warm-cycles, the lung tissues were removed and frozen in liquid nitrogen and stored at −80 °C. Lung tissues were homogenized in lysis buffer containing the protease inhibitors. Cytoplasmic and nuclear protein was extracted by nuclear and cytoplasmic extraction reagents kit (Applygen Technologies, Beijing, China) according to manufacturer’s instruction. The protein concentration was determined by MicroBCA (Pierce, Rockford, Illinois, USA). After electrophoresis on sodium dodecyl sulfate-polyacrylamide gels, the separated proteins were transferred to polyvinylidene difluoride (PVDF) membrane (Zerbrechlich-Fragile, Germany). Non-specific binding sites were blocked by pre-incubating PVDF membrane with 3% nonfat dried milk in Tris-buffered saline Tween (TBS-T). The PVDF membranes with target protein were incubated overnight at 4 °C with the primary antibodies against β-actin (1:4000), β-tubulin (1:2000), Occluding (1:1000), Claudin-5 (1:1000), JAM-1 (1:200), VE-cadherin (1:200), p-VE-cadherin (1:1000), ZO-1 (1:1000), Cav-1 (1:4000), phosphor-Cav-1 (1:1000), Src (1:1000), phosphor-Src (1:1000), NF-κB p65 (1:1000), Histone-3 (1:2000), Na+/K+-ATPase (1:200), NADPH oxidase subunit p47phox(1:200), p40phox (1:200) and p67phox (1:1000) in dilute buffer (3% nonfat dry milk and 0.1% TBS-T). After rinsing with TBS-T for 3 times, PVDF membranes were incubated with secondary antibody (1:4000, Cell Signaling Technology, Boston, VT, USA) at room temperature for 90 min and washed by TBS-T for 3 times. Antibody binding was detected by enhanced chemilucent detection system kit (Applygen Technologies, Beijing, China). Bands were visualized on X-ray film and the protein amount was estimated by quantifying the intensity of protein bands using Quantity one software (Bio-Rad, California, USA).

### Statistical analysis

All parameters were expressed as mean ± SE. Statistical analysis was performed using one-way ANOVA followed by Turkey test for multiple comparisons. A value of P less than 0.05 was considered to be statistically significant.

## Electronic supplementary material

Post-treatment with Ma-Huang-Tang ameliorates cold-warm-cycles induced rat lung injury

## References

[1] Kyobutungi C, Grau A, Stieglbauer G, Becher H (2005). Absolute temperature, temperature changes and stroke risk: a case-crossover study. European journal of epidemiology.

[2] Wolf K (2009). Air temperature and the occurrence of myocardial infarction in Augsburg, Germany. Circulation.

[3] Koskela HO (2007). Cold air-provoked respiratory symptoms: the mechanisms and management. International journal of circumpolar health.

[4] Mourtzoukou EG, Falagas ME (2007). Exposure to cold and respiratory tract infections. The international journal of tuberculosis and lung disease: the official journal of the International Union against Tuberculosis and Lung Disease.

[5] Lin S, Insaf TZ, Luo M, Hwang SA (2012). The effects of ambient temperature variation on respiratory hospitalizations in summer, New York State. International journal of occupational and environmental health.

[6] Currie, C. J. *et al.**Antibiotic treatment failure in four common infections in UK primary care 1991–2012: longitudinal analysis. BMJ* (*Clinical research ed.*) **349**, g5493, doi:10.1136/bmj.g5493 (2014). 10.1136/bmj.g5493 25249162

[7] Dicpinigaitis PV (2015). Clinical perspective-cough: an unmet need. Current opinion in pharmacology.

[8] Kim SY, Chang YJ, Cho HM, Hwang YW, Moon YS (2013). Non-steroidal anti-inflammatory drugs for the common cold. The Cochrane database of systematic reviews.

[9] Khadadah M, Mustafa S, Elgazzar A (2011). Effect of acute cold exposure on lung perfusion and tracheal smooth muscle contraction in rabbit. European journal of applied physiology.

[10] Davis MS (2007). Influx of neutrophils and persistence of cytokine expression in airways of horses after performing exercise while breathing cold air. American journal of veterinary research.

[11] Sabnis AS, Reilly CA, Veranth JM, Yost GS (2008). Increased transcription of cytokine genes in human lung epithelial cells through activation of a TRPM8 variant by cold temperatures. American journal of physiology. Lung cellular and molecular physiology.

[12] Jetha KA, Egginton S, Nash GB (2007). Changes in the integrin-mediated adhesion of human neutrophils in the cold and after rewarming. Biorheology.

[13] Nash GB, Abbitt KB, Tate K, Jetha KA, Egginton S (2001). Changes in the mechanical and adhesive behaviour of human neutrophils on cooling *in vitro*. Pflugers Archiv: European journal of physiology.

[14] Kubo T, Nishimura H (2007). Antipyretic effect of Mao-to, a Japanese herbal medicine, for treatment of type A influenza infection in children. Phytomedicine: international journal of phytotherapy and phytopharmacology.

[15] Wang HM, Lin SK, Yeh CH, Lai JN (2014). Prescription pattern of Chinese herbal products for adult-onset asthma in Taiwan: a population-based study. Annals of allergy, asthma & immunology: official publication of the American College of Allergy, Asthma, & Immunology.

[16] Ma CH, Ma ZQ, Fu Q, Ma SP (2014). Ma Huang Tang ameliorates asthma though modulation of Th1/Th2 cytokines and inhibition of Th17 cells in ovalbumin-sensitized mice. Chinese journal of natural medicines.

[17] Nagai T (2014). Alleviative Effects of a Kampo (a Japanese Herbal) Medicine “Maoto (Ma-Huang-Tang)” on the Early Phase of Influenza Virus Infection and Its Possible Mode of Action. Evidence-based complementary and alternative medicine: eCAM.

[18] Sun, Y., Hu, G., Zhang, X. & Minshall, R. D. Phosphorylation of caveolin-1 regulates oxidant-induced pulmonary vascular permeability via paracellular and transcellular pathways. *Circulation research* **105**, 676–685, 615 p following 685, doi: 10.1161/CIRCRESAHA.109.201673 (2009). 10.1161/CIRCRESAHA.109.201673 PMC2776728 19713536

[19] Hu G, Vogel SM, Schwartz DE, Malik AB, Minshall RD (2008). Intercellular adhesion molecule-1-dependent neutrophil adhesion to endothelial cells induces caveolae-mediated pulmonary vascular hyperpermeability. Circulation research.

[20] Lee IT, Yang CM (2013). Inflammatory signalings involved in airway and pulmonary diseases. Mediators of inflammation.

[21] Bao Z (2009). A novel antiinflammatory role for andrographolide in asthma via inhibition of the nuclear factor-kappaB pathway. American journal of respiratory and critical care medicine.

[22] Lee I, Dodia C, Chatterjee S, Feinstein SI, Fisher AB (2014). Protection against LPS-induced acute lung injury by a mechanism-based inhibitor of NADPH oxidase (type 2). American journal of physiology. Lung cellular and molecular physiology.

[23] Kulka JP (1961). Vasomotor microcirculatory insufficiency: observations on nonfreezing cold injury of the mouse ear. Angiology.

[24] Zook, N*.**et al**.* Microcirculatory studies of frostbite injury. *Annals of plastic surgery* **40**, 246–253, discussion 254–245 (1998). 10.1097/00000637-199803000-00009 9523607

[25] Chao J, Wood JG, Gonzalez NC (2009). Alveolar hypoxia, alveolar macrophages, and systemic inflammation. Respiratory research.

[26] Frank PG, Woodman SE, Park DS, Lisanti MP (2003). Caveolin, caveolae, and endothelial cell function. Arteriosclerosis, thrombosis, and vascular biology.

[27] Furuse M (2010). Molecular basis of the core structure of tight junctions. Cold Spring Harbor perspectives in biology.

[28] Sverdlov M, Shajahan AN, Minshall RD (2007). Tyrosine phosphorylation-dependence of caveolae-mediated endocytosis. Journal of cellular and molecular medicine.

[29] Song H (2014). Reduction of brain barrier tight junctional proteins by lead exposure: role of activation of nonreceptor tyrosine kinase Src via chaperon GRP78. Toxicological sciences: an official journal of the Society of Toxicology.

[30] Hardyman MA (2013). TNF-alpha-mediated bronchial barrier disruption and regulation by src-family kinase activation. The Journal of allergy and clinical immunology.

[31] Ushio-Fukai M (2009). Compartmentalization of redox signaling through NADPH oxidase-derived ROS. Antioxidants & redox signaling.

[32] Lee HS (2007). Src tyrosine kinases mediate activations of NF-kappaB and integrin signal during lipopolysaccharide-induced acute lung injury. Journal of immunology.

[33] Gao Z (2005). Coactivators and corepressors of NF-kappaB in IkappaB alpha gene promoter. The Journal of biological chemistry.

[34] Yang N (2014). Pretreatment with andrographolide pills((R)) attenuates lipopolysaccharide-induced pulmonary microcirculatory disturbance and acute lung injury in rats. Microcirculation (New York, N.Y.: 1994).

[35] Ma LQ (2014). Posttreatment with Ma-Xing-Shi-Gan-Tang, a Chinese medicine formula, ameliorates lipopolysaccharide-induced lung microvessel hyperpermeability and inflammatory reaction in rat. Microcirculation (New York, N.Y.: 1994).

[36] Lin F (2013). Salvianolic Acid B protects from pulmonary microcirculation disturbance induced by lipopolysaccharide in rat. Shock (Augusta, Ga.).

[37] Zhang Y (2014). Ginsenoside Rb1 ameliorates lipopolysaccharide-induced albumin leakage from rat mesenteric venules by intervening in both trans- and paracellular pathway. American journal of physiology. Gastrointestinal and liver physiology.

