# Supplementary material for: Post-treatment with Ma-Huang-Tang ameliorates cold-warm-cycles induced rat lung injury
Source: Sci Rep. 2017 Mar 22;7:312. doi: 10.1038/s41598-017-00459-3 (PMC5428516; doi:10.1038/s41598-017-00459-3)
Supplement: Supplementary file 1 — Post-treatment with Ma-Huang-Tang ameliorates cold-warm-cycles induced rat lung injury [file 41598_2017_459_MOESM1_ESM.doc]

**Post-treatment with Ma-Huang-Tang ameliorates cold-warm-cycles induced rat lung injury**

Meng-Meng Xiao; Chun-Shui Pan; Yu-Ying Liu; Li-Qian Ma; Li Yan; Jing-Yu Fan; Chuan-She Wang; Rong Huang; Jing-Yan Han

**Supplementary Materials:**

Materials and Methods

Tables S1

Figures S1-S10

**Materials and Methods:**

1. Animals.

Male Sprague-Dawley rats weighing 180-200 g were obtained from the Animal Center of Peking University Health Science Center. The certificate code number was SCXK 2006-0008. The animals were housed at 24 ± 1 °C and relative humidity of 50 ± 1% with a 12 h light/dark cycle and given standard laboratory diet and water. The animals were fasted for 12 h before experiment but free to access to water. The experimental procedures were in accordance with the European commission guideline (2010/63/EU). All animals were handled according to the guidelines of the Peking University Animal Research Committee. The experimental protocol was approved by the Committee on the Ethics of Animal Experiments of Peking University Health Science Center (LA2015143).

2. Agents

MHT granules, consisted of HE granule (39.4%), RC granule (26.3%), SAA granule (15.5%) and RG granule (18.8%), were provided by Guangdong Yi Fang Pharmaceutical Co Ltd (Guangzhou, China). Rhodamine 6G was from Fluka Chemie AG (Buchs, Switzerland). Antibodies against p-VE-cadherin，β-actin and Histone-3 were purchased from Cell Signaling Technology (Beverly, MA, USA). Antibodies against NF-κB p65, Na+/K+-ATPase, NADPH oxidase subunit p47phox and p40phox, VE-cadherin were purchased from Santa Cruz Biotechnology (Santa Cruz, CA, USA). Antibodies against NADPH oxidase subunit p67phox were purchased from Abcam (Cambridge, UK).

3. Experiment protocols.

Rats were randomly assigned into one of eight groups: (1) Control group, (2) Post-cold 6h group, (3) Post-cold 24h group, (4) Post-cold 24h + MHT group, (5) Post-cold 24h + HE group, (6) Post-cold 24h + RC group, (7) Post-cold 24h + SAA group, (8) Post-cold 24h + RG group, 8 in each. The rats were exposed to a cold environment (-15 ℃) for 1 h and then shifted to a warm environment (25 ℃) for 30 min. This cold-warm alternation cycled 4 times to imitate environment temperature alternation, and then the rats were maintained in room temperature. Six hours later, the rats were subjected to determination of various parameters (Post-cold 6 h group) or administrated by gavage with MHT suspension (1.87 g/kg, Post-cold 24 h + MHT group), HE suspension (0.735 g/kg, Post-cold 24 h + HE group), RC suspension (0.490 g/kg, Post-cold 24 h +RC group), SAA suspension (0.291 g/kg, Post-cold 24 h +SAA group), and RG suspension (0.350 g/kg, Post-cold 24 h +RG group), and subjected to determination of parameters 24 h after transferring to room temperature. Rats of Control groups were kept at 25 ℃ for 6 h without food and water delivered, and then transferred to room temperature, administrated 6 h later by gavage with normal saline (Control group). All of the parameters of the rats in Control groups were determined 24 h after transferring to room temperature. The techniques for histology, western blot and statistics were exactly identical to those used in the main body of the paper.

4. Histological and immunohistochemical staining.

Histologic and immunohistochemical examinations were carried out as previously described [^35-37^](#_ENREF_35). In brief, 6 or 24 h after cold-warm-cycles, rat right middle lung lobe was excised, fixed in 4% paraformaldehyde in 0.1 M phosphate buffer solution (pH 7.4), and processed for paraffin sections using an automated processing unit (RM2255, Leica, Berlin, Germany). The sections of 5 μm of pulmonary specimens for histology were stained by hematoxylin and eosin (H&E). The images were captured by a digital camera connected to a microscope (BX512DP70, Olympus, Tokyo, Japan). The sections for immunohistochemistry were incubated with rabbit polyclonal antibody against MPO (1:200, Thermo Scientific, Fremont, CA, USA), rabbit antibody against CD68 (1:50, Abcam, Cambridge, UK) or mouse antibody against Claudin-5 (1:50, Santa Cruz Biotechnology, Santa Cruz, USA) after blocked with bovine serum albumin, and then incubated with a biotinylated secondary antibody followed by an avidin-biotin-complex-peroxidase kit or a fluorescent secondary antibody. Positive staining was revealed by reacting with diaminobenzidine (BD Biosciences Pharmingen, CA, USA) or a laser scanning confocal microscope (TCS SP5, Leica, Mannheim, Germany).

5. Western blotting assay.

Rats were sacrificed 6 or 24 h after cold-warm-cycles, the lung tissues were removed and frozen in liquid nitrogen and stored at -80 °C. Lung tissues were homogenized in lysis buffer containing the protease inhibitors. Cytoplasmic and nuclear protein was extracted by nuclear and cytoplasmic extraction reagents kit (Applygen Technologies, Beijing, China) according to manufacturer’s instruction. The protein concentration was determined by MicroBCA (Pierce, Rockford, Illinois, USA). After electrophoresis on sodium dodecyl sulfate-polyacrylamide gels, the separated proteins were transferred to polyvinylidene difluoride (PVDF) membrane (Zerbrechlich-Fragile, Germany). Non-specific binding sites were blocked by pre-incubating PVDF membrane with 3% nonfat dried milk in Tris-buffered saline Tween (TBS-T). The PVDF membranes with target protein were incubated overnight at 4 °C with the primary antibodies against β-actin (1:4000), VE-cadherin (1:200), p-VE-cadherin (1:1000), NF-κB p65 (1:1000), Histone-3 (1:2000), Na+/K+-ATPase (1:200), NADPH oxidase subunit p47phox(1:200), p40phox (1:200) and p67phox (1:1000) in dilute buffer (3% nonfat dry milk and 0.1 % TBS-T). After rinsing with TBS-T for 3 times, PVDF membranes were incubated with secondary antibody (1:4000, Cell Signaling Technology, Boston, VT, USA) at room temperature for 90 min and washed by TBS-T for 3 times. Antibody binding was detected by enhanced chemilucent detection system kit (Applygen Technologies, Beijing, China). Bands were visualized on X-ray film and the protein amount was estimated by quantifying the intensity of protein bands using Quantity one software (Bio-Rad, California, USA).

**Tables S1**

**Summary of the effect and efficiency comparison of MHT and its components on cold-warm-cycles-induced alterations in lung tissue.**

|  | MHT | HE | RC | SAA | RG |
| --- | --- | --- | --- | --- | --- |
| NF-κB p65 cytosolic fraction | ↑* | ↑* | ↑*# | — # | — # |
| NF-κB p65 nuclear fraction | ↓* | ↓* | ↓*# | — # | — # |
| p40phox  cytosolic fraction | ↑* | ↑* | ↑* | ↑* | — # |
| p40phox  membrane fraction | ↓* | ↓*# | ↓*# | —# | —  # |
| p47phox  cytosolic fraction | ↑* | ↑* | ↑* | ↑* | — # |
| p47phox  membrane fraction | ↓* | ↓*# | ↓*# | — # | — # |
| p67phox  cytosolic fraction | ↑* | ↑* | ↑* | ↑* | — # |
| p67phox  membrane fraction | ↓* | ↓* | ↓* | — # | — # |

HE, herba ephedrae ; RC, ramulus cinnamomi; SAA, semen armeniacae amarumon ; RG, radix glycyrrhizae. *p<0.05 vs. Post-cold 24h, #p<0.05 vs. Post-cold 24h+MHT. ↓, decrease; ↑, increase；—, no change.

**Figure S1**


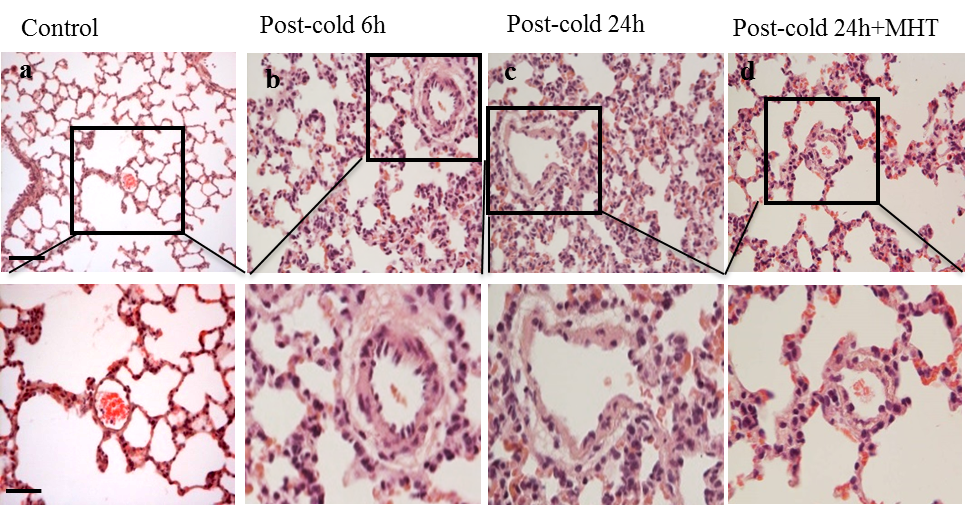


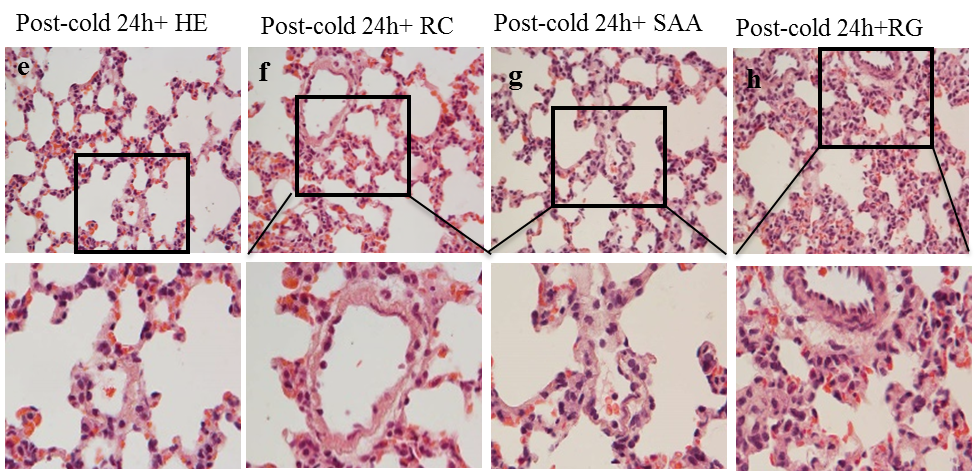


**Figure S1.** Effects of post-treatment with MHT and its components herba ephedrae (HE), ramulus cinnamomi (RC), semen armeniacae amarum (SAA) and radix glycyrrhizae (RG) on histology of the lung tissue. Shown are representative H&E staining images of rat lung tissue in different groups. Bar=100 μm. The area within the rectangle in each image in upper panel is enlarged and presented below. Bar=50 μm.

**Figure S2**


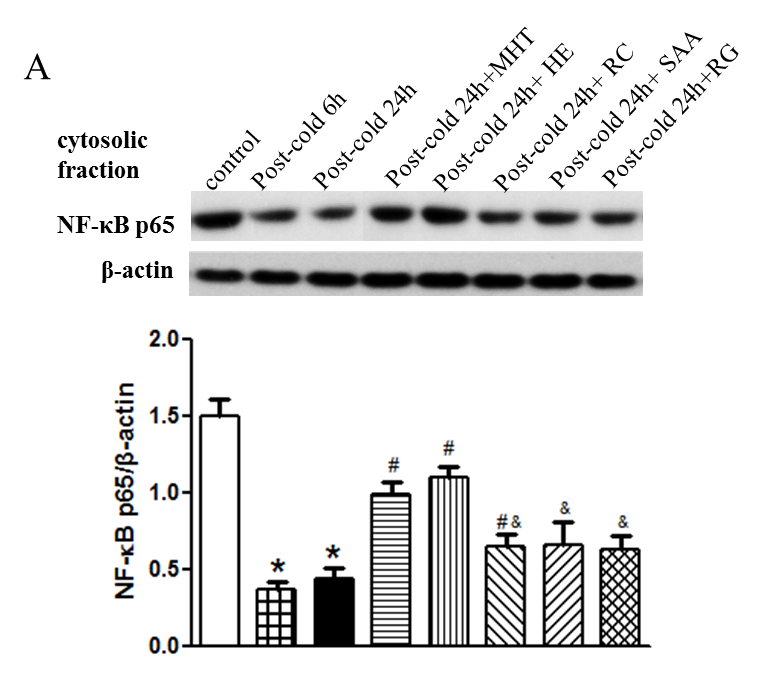

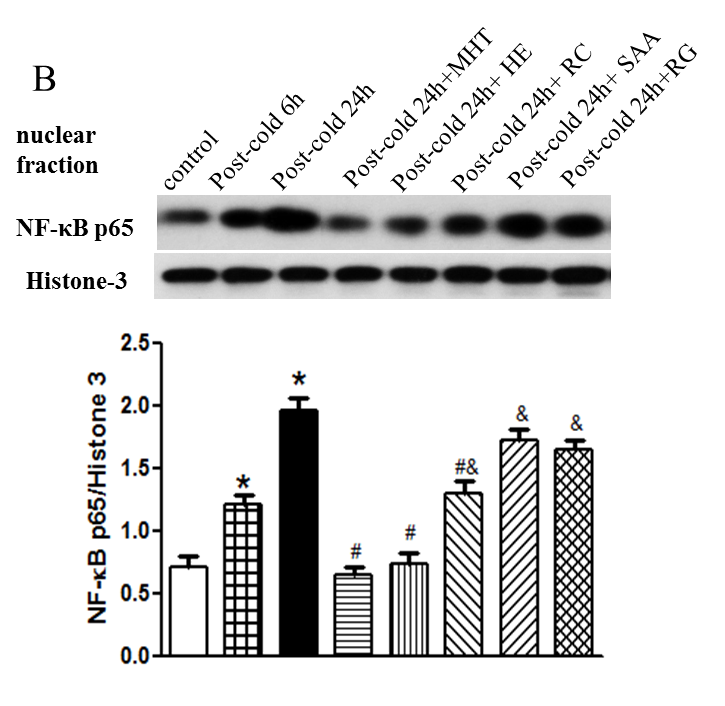


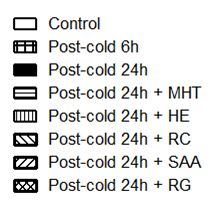

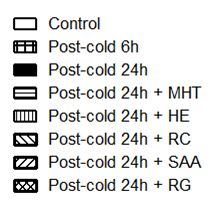


**Figure S2**. Effects of post-treatment with MHT and its HE, RC, SAA and RG on nuclear translocation of NF-κB p65. **A:** The expression and quantification of NF-κB p65 in cytosolic fraction; **B:** The expression and quantification of NF-κB p65 in nuclear fraction. N=4. Results are presented as mean ± SE. *P <0.05 vs. Control group; # P <0.05 vs. Post-cold 24h group; & P <0.05 vs. Post-cold 24h + MHC group.

**Figure S3**


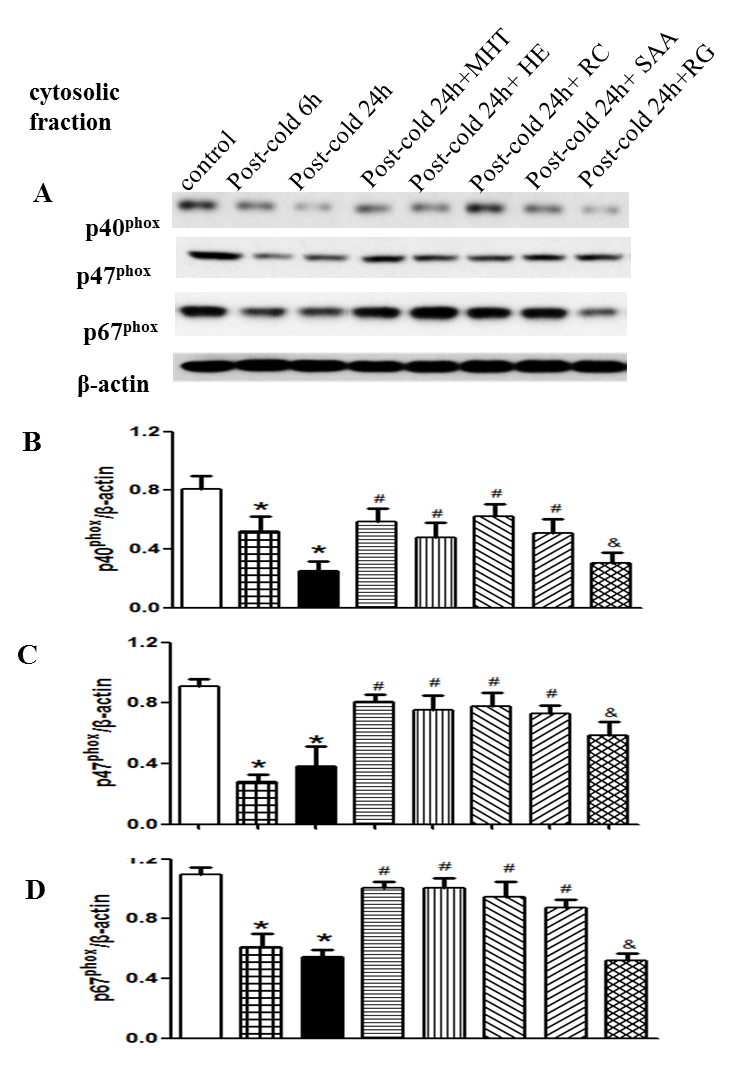

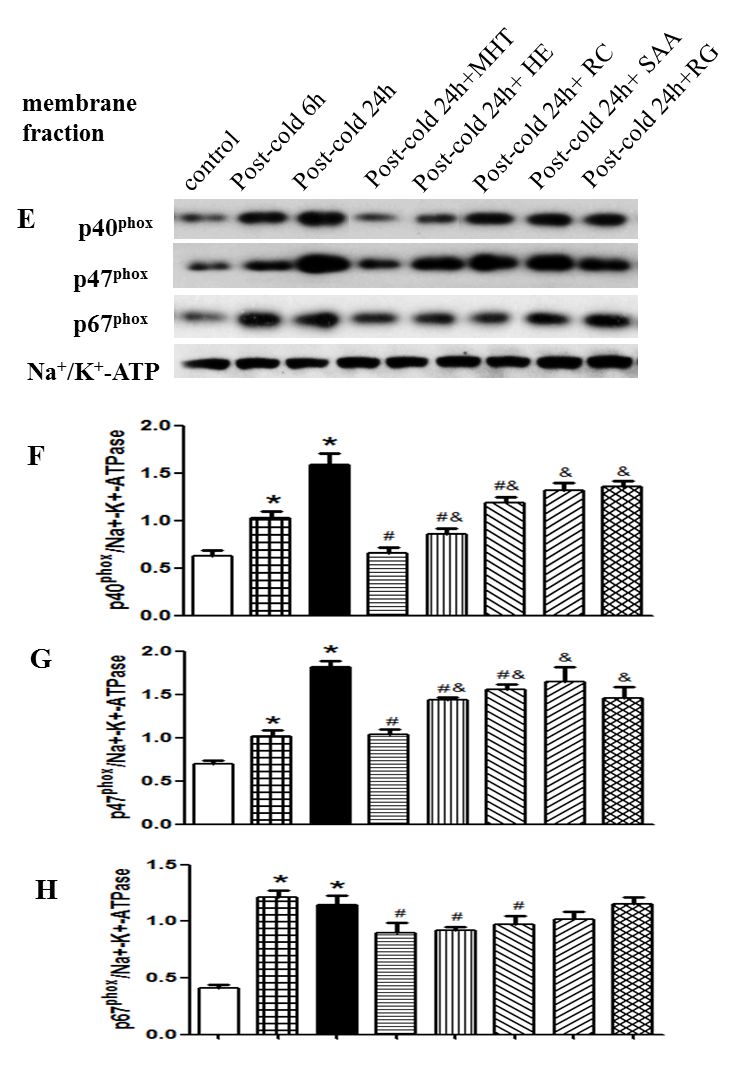


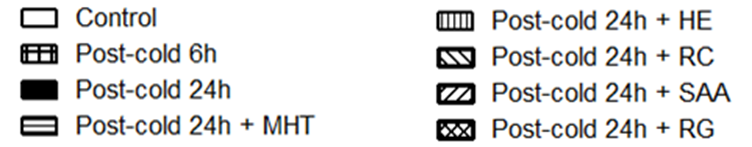


**Figure S3.** Effects of post-treatment with MHT and its components- HE, RC, SAA and RG on membrane translocation of NADPH oxidase subunits p40^phox^, p47^phox^ and p67^phox^. **A, E:** The representative western blot bands of NADPH oxidase subunits p40^phox^, p47^phox^ and p67^phox^ in cytosolic fraction and membrane fraction, respectively; **B:** Quantification of p40^phox^ in cytosol; **C:** Quantification of p47^phox^ in cytosol; **D:** Quantification of p67^phox^ in cytosol; **F:** Quantification of p40^phox^ in cell membrane; **G:** Quantification of p47^phox^ in cell membrane; **H:** Quantification of p67^phox^ in cell membrane; N=4. Results are presented as mean ± SE. *P <0.05 vs. Control group; # P <0.05 vs. Post-cold 24 h group. & P <0.05 vs. Post-cold 24h + MHC group.

**Figure S4**


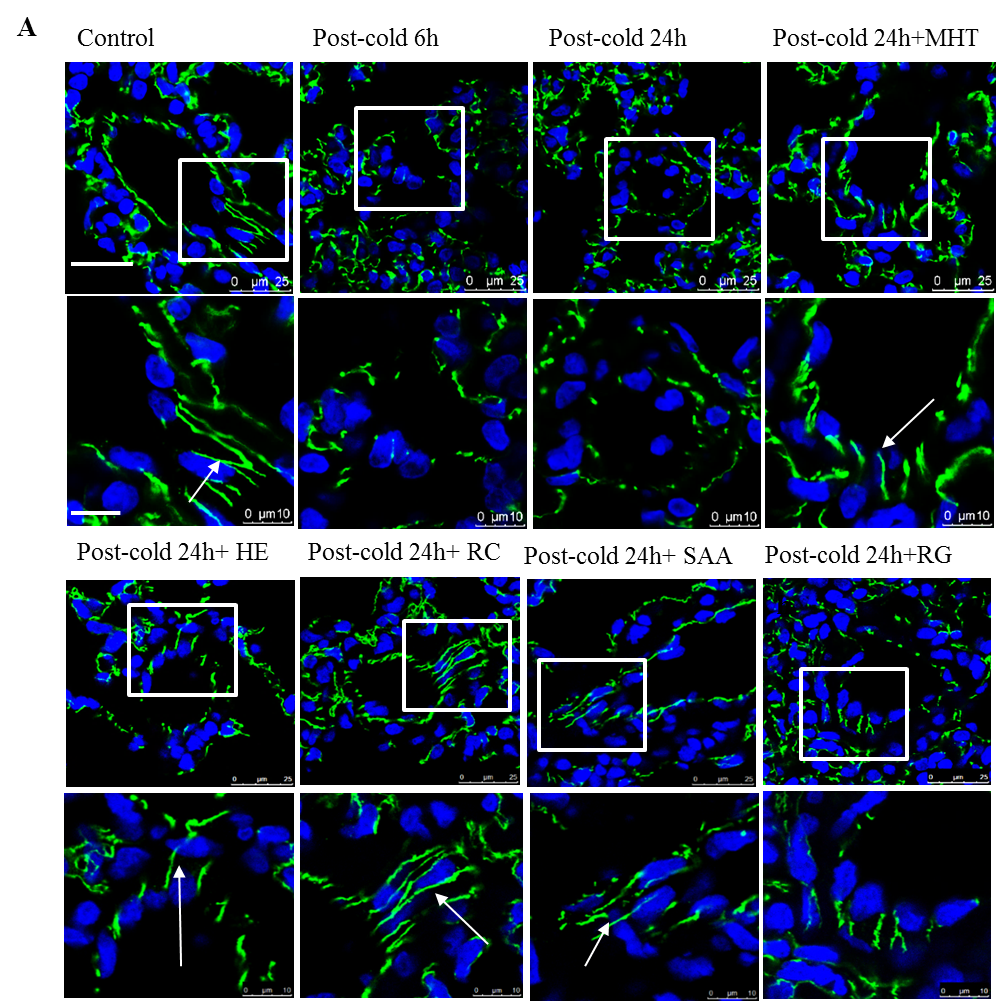


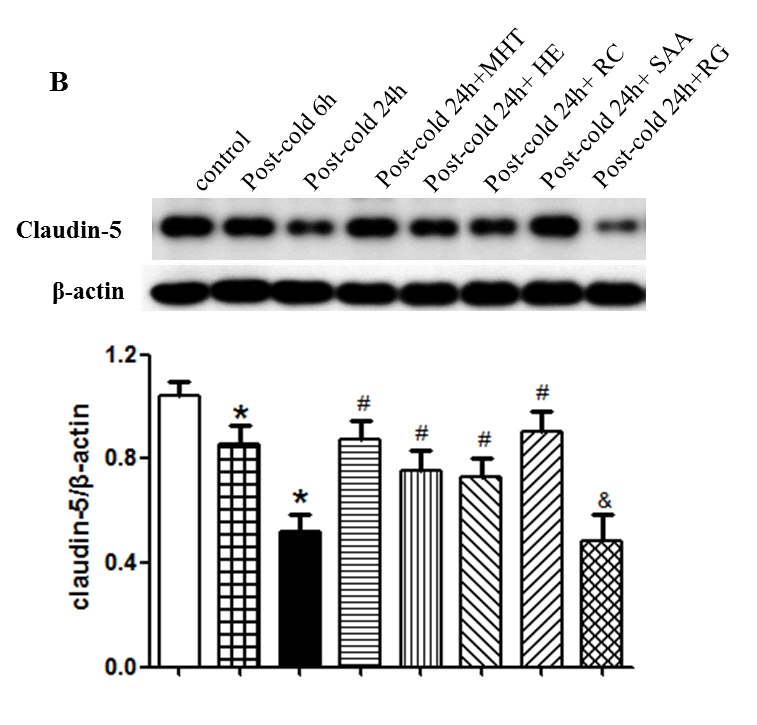


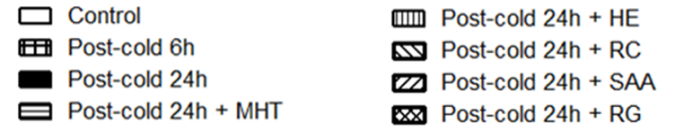


**Figure S4.** Effects of post-treatment with MHT and its components herba ephedrae (HE), ramulus cinnamomi (RC), semen armeniacae amarum (SAA) and radix glycyrrhizae (RG) on distribution and expression of claudin-5. A: Representative immunofluorescence confocal images of rat lung tissue in various groups. Bar=25 μm. The area within the rectangle in each image in upper panel is enlarged and presented below. Bar=10 μm. The sections were immunochemically stained for Claudin-5 (green). B: The expression and quantification of claudin-5 in lung tissue. N=4. Results are presented as mean ± SE. *P <0.05 vs. Control group; # P <0.05 vs. Post-cold 24h group; & P <0.05 vs. Post-cold 24h + MHC group.

**Figure S5**


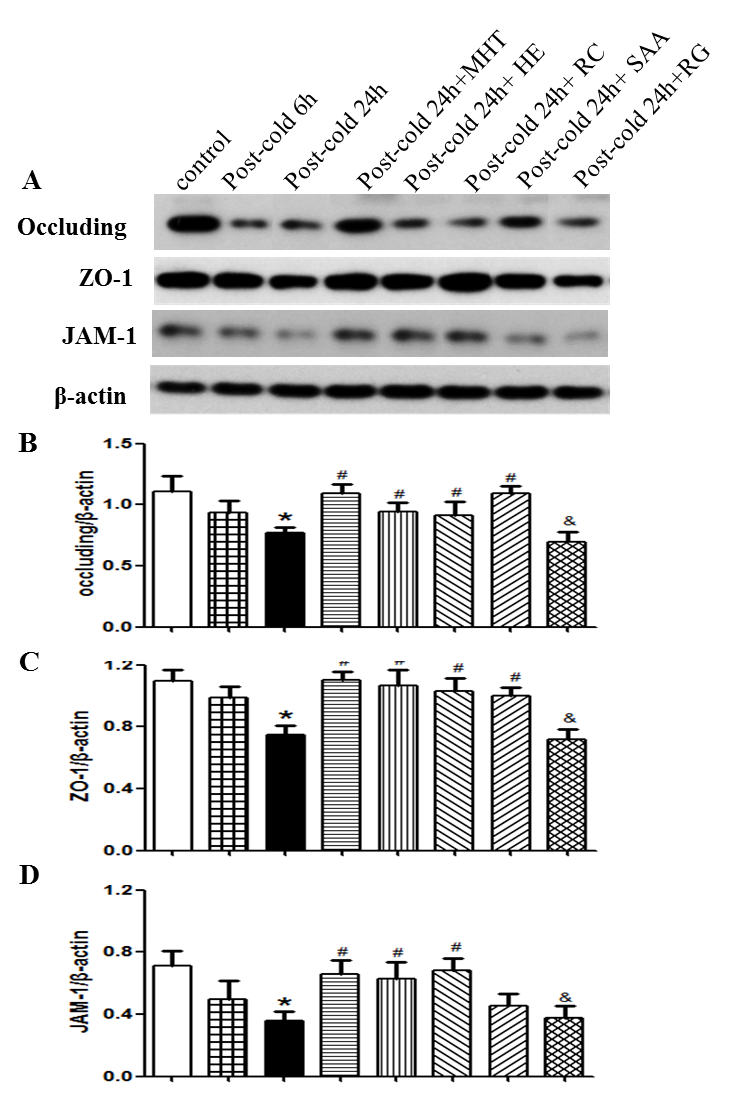


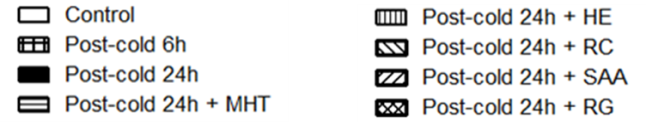


**Figure S5.** Effects of post-treatment with MHT and its HE, RC, SAA and RG on expressions of tight junction proteins. A: The representative western blot bands of tight junction proteins occluding, ZO-1 and JAM-1; B: The quantification of Occluding in lung tissue; C: The quantification of ZO-1 in lung tissue; D: The quantification of JAM-1 in lung tissue. N=4. Results are presented as mean ± SE. *P <0.05 vs. Control group; # P <0.05 vs. Post-cold 24h group; & P <0.05 vs. Post-cold 24h + MHC group.

**Figure S6**


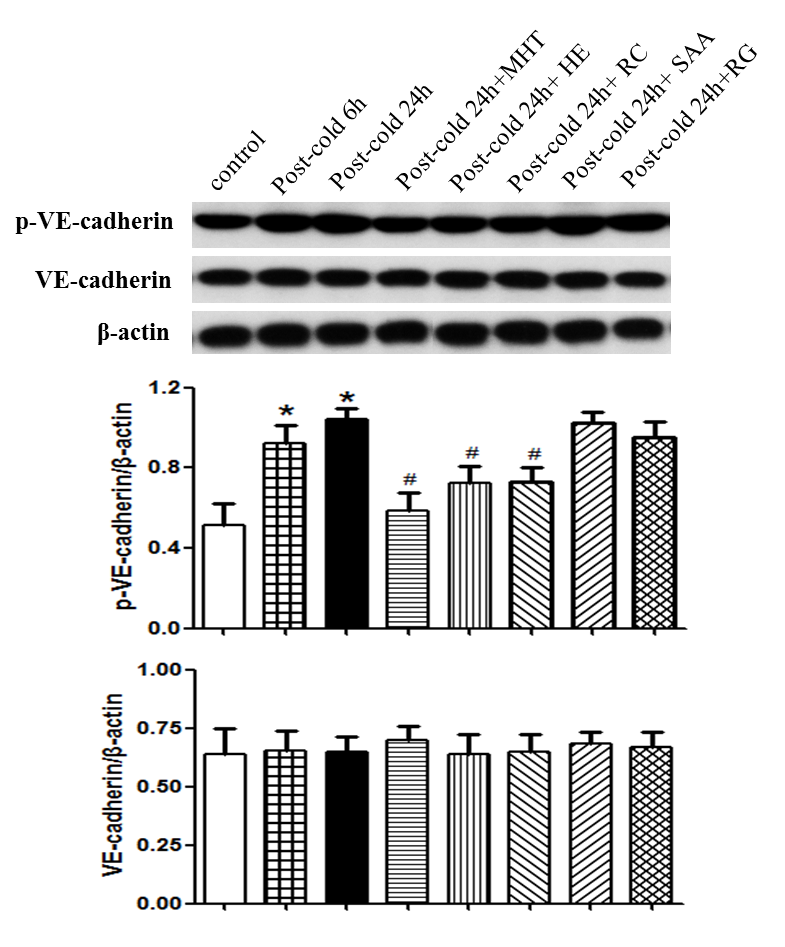


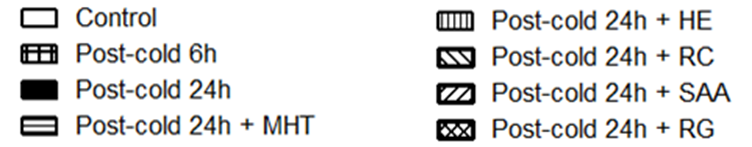


**Figure S6.** Effects of post-treatment with MHT and its HE, RC, SAA and RG on phosphorylation of VE-cadherin. A: The representative western blot bands of VE-cadherin and phosphorylation of VE-cadherin; B: The quantification of phosphorylation of VE-cadherin in lung tissue; C: The quantification of VE-cadherin in lung tissue; N=4. Results are presented as mean ± SE. *P <0.05 vs. Control group; # P <0.05 vs. Post-cold 24h group; & P <0.05 vs. Post-cold 24h + MHC group.

**Figure S7**


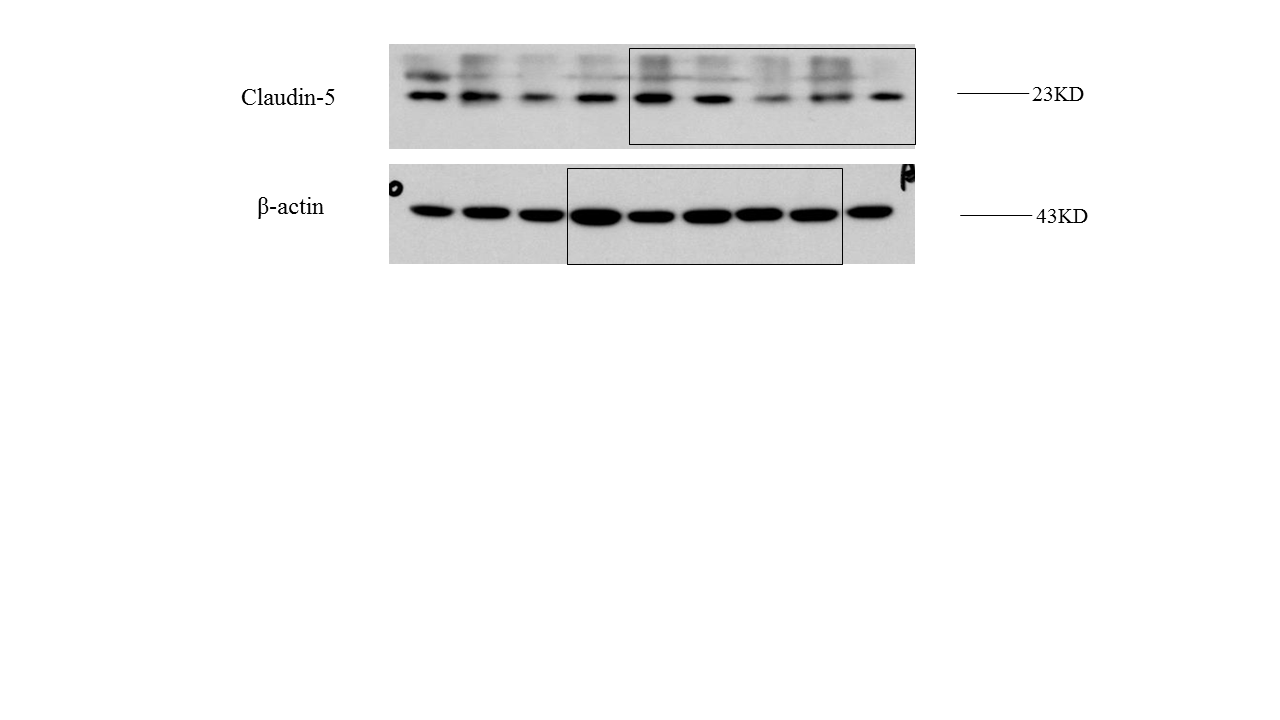


**Figure S7.** The full-length representative western blotting bands of Claudin-5 and β-actin in different groups as shown with indication of molecular size. Selected fractions of bands used in the main text were framed as shown in the figure.

**Figure S8**


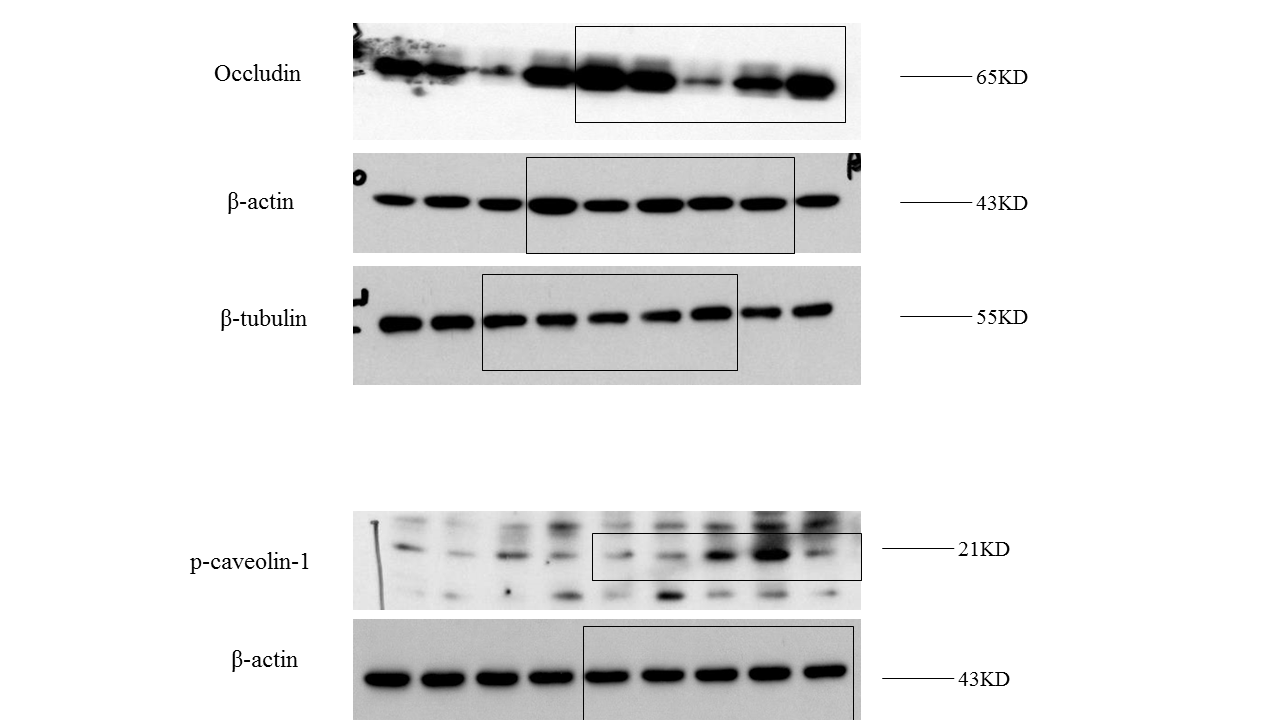


**Figure S8.** The full-length representative western blotting bands of Occluding, β-actin,β-tubulin and p-caveolin-1 in different groups as shown with indication of molecular size. Selected fractions of bands used in the main text were framed as shown in the figure.

**Figure S9**


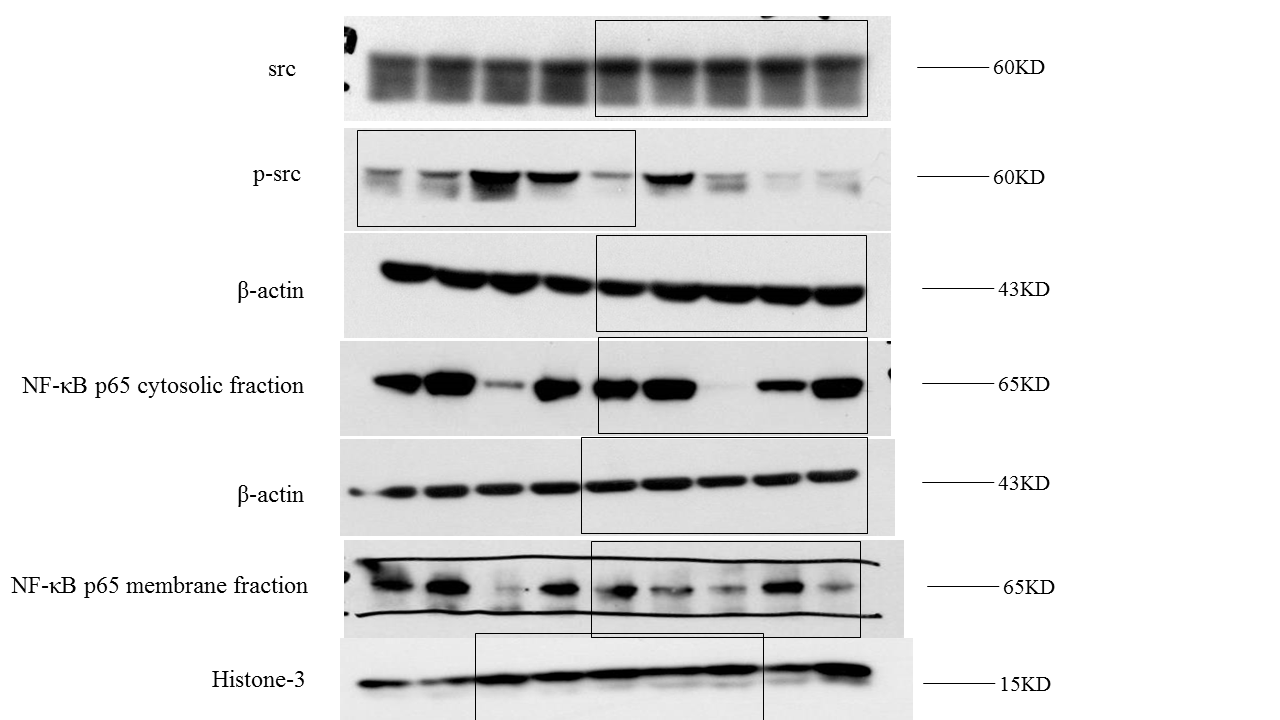


**Figure S9.** The full-length representative western blotting bands of src，p-src，β-actin, NF-κB and Histone-3 in different groups as shown with indication of molecular size. Selected fractions of bands used in the main text were framed as shown in the figure.

**Figure S10**


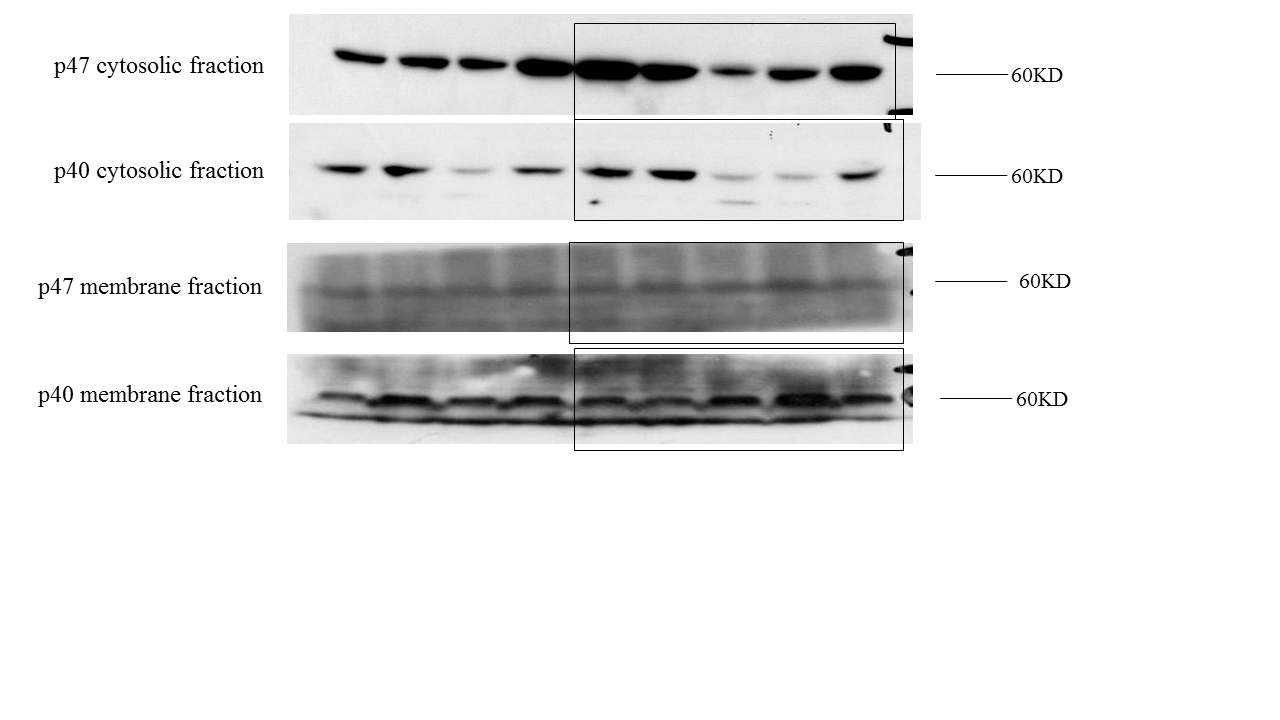


**Figure S10.** The full-length representative western blotting bands of p47^phox^ and p40^phox^ of cytosolic and membrane fraction in different groups as shown with indication of molecular size. Selected fractions of bands used in the main text were framed as shown in the figure.
